# Late symptoms in long-term gynaecological cancer survivors after radiation therapy: a population-based cohort study

**DOI:** 10.1038/bjc.2011.315

**Published:** 2011-08-16

**Authors:** H Lind, A-C Waldenström, G Dunberger, M al-Abany, E Alevronta, K-A Johansson, C Olsson, T Nyberg, U Wilderäng, G Steineck, E Åvall-Lundqvist

**Affiliations:** 1 Clinical Cancer Epidemiology, Department of Oncology and Pathology, Karolinska Institutet, Stockholm, Sweden; 2 Division of Clinical Cancer Epidemiology, Department of Oncology, Institute of Clinical Sciences, Sahlgrenska Academy at University of Gothenburg, Gothenburg, Sweden; 3 Department of Oncology, Sahlgrenska University Hospital, Gothenburg, Sweden; 4 Department of Medical Physics, Karolinska University Hospital, Stockholm, Sweden; 5 Department of Radiation Physics, Institute of Clinical Sciences, Sahlgrenska Academy at University of Gothenburg, Gothenburg, Sweden; 6 Department of Radiation Physics, Institute of Clinical Sciences, Sahlgrenska Academy at University of Gothenburg, Gothenburg, Sweden; 7 Department of Gynecologic Oncology, Karolinska University Hospital, Stockholm, Sweden

**Keywords:** gynaecological cancer survivors, radiation therapy, long-term side-effects, pelvic

## Abstract

**Background:**

We surveyed the occurrence of physical symptoms among long-term gynaecological cancer survivors after pelvic radiation therapy, and compared with population-based control women.

**Methods:**

We identified a cohort of 789 eligible gynaecological cancer survivors treated with pelvic radiation therapy alone or combined with surgery in Stockholm or Gothenburg, Sweden. A control group of 478 women was randomly sampled from the Swedish Population Registry. Data were collected through a study-specific validated postal questionnaire with 351 questions concerning gastrointestinal and urinary tract function, lymph oedema, pelvic bones and sexuality. Clinical characteristics and treatment details were retrieved from medical records.

**Results:**

Participation rate was 78% for gynaecological cancer survivors and 72% for control women. Median follow-up time after treatment was 74 months. Cancer survivors reported a higher occurrence of symptoms from all organs studied. The highest age-adjusted relative risk (RR) was found for emptying of all stools into clothing without forewarning (RR 12.7), defaecation urgency (RR 5.7), difficulty feeling the need to empty the bladder (RR 2.8), protracted genital pain (RR 5.0), pubic pain when walking indoors (RR 4.9) and erysipelas on abdomen or legs at least once during the past 6 months (RR 3.6). Survivors treated with radiation therapy alone showed in general higher rates of symptoms.

**Conclusion:**

Gynaecological cancer survivors previously treated with pelvic radiation report a higher occurrence of symptoms from the urinary and gastrointestinal tract as well as lymph oedema, sexual dysfunction and pelvic pain compared with non-irradiated control women. Health-care providers need to actively ask patients about specific symptoms in order to provide proper diagnostic investigations and management.

With increasing numbers of long-term gynaecological cancer survivors, the prevention and alleviation of late side effects after treatment have become a priority. A significant number of these survivors have been treated with pelvic radiation therapy. Radiation therapy may induce pathophysiological changes in all normal tissue or organs within the irradiated volume, which in some cases will lead to symptoms negatively affecting daily activities and quality of life. Although some late effects from normal tissues have been extensively explored (Eifel *et al*, 1995; Grigsby *et al*, 1995; Creutzberg *et al*, 2001; Nout *et al*, 2009), others have been less characterised (Bentzen *et al*, 2010).

The mechanism leading to radiotherapy-induced late side effects is multifactorial. Irradiation may induce direct cell killing through DNA double-strand breaks. However, the interaction of ionising radiation with normal tissue also induces activation of cytokines and growth factors leading to a risk of fibrosis with decreased elasticity and compliance and increased risk of strictures. In addition, impaired function in blood- and lymph vessels along with neural damage may enhance the effect (Denham and Hauer-Jensen, 2002; Bentzen, 2006; Hauer-Jensen *et al*, 2007). Severe acute side effects have also been associated with consequential late effects in normal tissue (Dörr and Hendry, 2001). Together with patient characteristics, such as smoking, co-morbidities and genetic factors, these radiation-induced effects may lead to changes affecting specific physiological functions (Andreyev, 2007; Bentzen *et al*, 2010).

Radiation oncologists have a long tradition of recording late toxicity after cancer treatment. Several instruments exist, for example, the Franco-Italian glossary (Sinistrero *et al*, 1993), the RTOG/EORTC late radiation morbidity scoring schema (Rubin *et al*, 1995), the SOMA-LENT system (Mornex *et al*, 1997) and CTCAE (Trotti *et al*, 2003), but many of these combine multiple signs and symptoms into a single grade leading to loss of specificity. Only rarely have the toxicity scales been developed with guidance from the survivors. In addition, the great variety of study designs and measures of quality of life used in studies of gynaecological cancer survivors may make comparisons difficult. Although many of the questionnaires commonly used in clinical trials have been validated, for example, the FACT (Cella *et al*, 1993) and EORTC QLQ (Sprangers *et al*, 1993) questionnaires, these do not explore the impact of each specific symptom on daily activities (Steineck *et al*, 2002).

Personal identity numbers and official population-based registers in Sweden covering all Swedish citizens offer exceptionally good conditions for studying cancer survivors without selection-induced problems. We performed a population-based cohort study on gynaecological cancer survivors concerning late symptoms after pelvic radiation therapy. We used a validated study-specific questionnaire in order to cover specific physical symptoms, sexuality, psychological dimensions and their impact on social functioning since no standardised existing instruments measure all these aspects. Here, we present self-reported symptoms from irradiated normal tissues, that is, the anal sphincter, the bowel, the urinary tract, the pelvic bones, the lower abdomen, legs and symptoms related to sexuality among gynaecological cancer survivors and compare their occurrence with that of population-based control women. In addition, we present a detailed description of the demographic and clinical characteristics and how therapy was delivered in order to provide a background for coming publications.

## Materials and methods

### Study population

**Figure 1:**
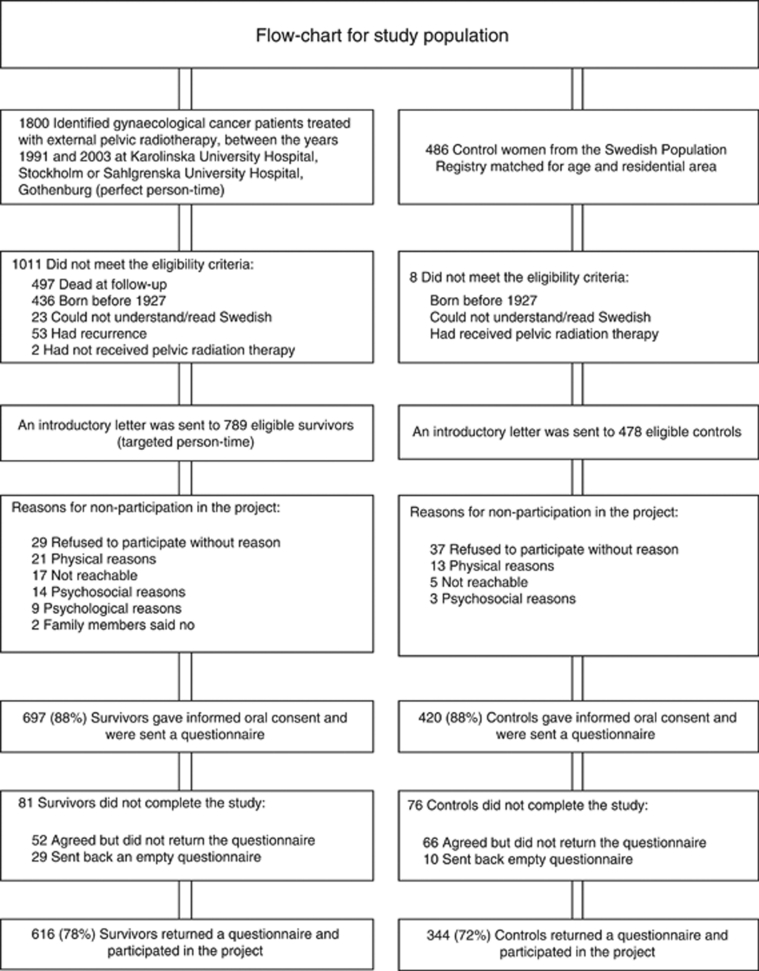
Flow chart for study population.

A cohort of 1800 women treated with external pelvic radiation therapy alone or as part of combination therapy for a gynaecological malignancy was identified. The women were consecutively treated between February 1991 and December 2003 at Radiumhemmet, Karolinska University Hospital in Stockholm or at Jubileumskliniken, Sahlgrenska University Hospital in Gothenburg (Figure 1). At follow-up in January 2006, 789 survivors (Stockholm *n*=595 and Gothenburg *n*=194) met the eligibility criteria, that is, born 1927 or later, being able to read and understand Swedish, and having no recurrent disease. Eligible survivors received an introductory letter. Medical records were reviewed to confirm the cancer diagnosis, stage of disease and treatment techniques regarding surgery, radiation therapy and chemotherapy.

As controls, we recruited 478 women from the Swedish Population Register, matched for age and residency and who had not had pelvic radiation therapy (Figure 1). An error in the matching procedure led to a younger control population (median age 57.5) compared with the cancer survivors (median age 66.0), which was adjusted for in the statistical analyses. The Regional Ethics Committee at the Karolinska Institute approved the study.

### Questionnaire

Our methods for studies of cancer survivorship and development of study-specific questionnaires have been documented in >80 scientific papers (Bergmark *et al*, 1999; Steineck *et al*, 2006). A description of the development and validation of the present questionnaire has previously been reported (Dunberger *et al*, 2010). In summary, the questionnaire was developed during an 18 months long qualitative phase including semi-structured interviews with 26 gynaecological cancer survivors previously treated with pelvic radiation therapy. A study-specific questionnaire was constructed consisting of 351 questions covering symptoms from the gastrointestinal tract, urinary bladder, genitals, pelvic bones, abdomen and legs. Questions concerning demographics, sexual function, intercurrent diseases, psychological and quality-of-life issues were included. In each part of the questionnaire, we asked about the incidence, prevalence, intensity and duration of the symptoms and their impact on different aspects of social functioning.

Face-to-face validation of the final version was made to ensure that it was conceivable and had satisfactory internal consistency. Participation rate and rate of missing values were tested in a pilot study. The main study, the quantitative phase, was carried out during January–October 2006. Eligible women who gave informed consent received a postal questionnaire. Confidentiality was maintained by numbering the questionnaires.

### Radiation therapy

Treatment was administered according to local treatment programmes and applied study protocols that were ongoing at the time of treatment. External beam radiation therapy (EBRT) was based on computed tomography (CT) scans performed before radiation therapy. Computed tomography scans were made in treatment position on a flat table top, using laser markers and conversion factors to electron density. Computed tomography slices ranged from 5 to 20 mm. The EBRT dose was prescribed either at isocenter or as mean dose to the target covering at least 95% of the planning target volume (ICRU, 1993). Patients were treated in supine position, using linear accelerators or a racetrack accelerator with two opposing fields or a four-field box technique. Daily dose per fraction varied between 1.6 and 2.0 Gy. External beam radiation therapy was verified by portal image films and with check-and-confirm systems. Brachytherapy (BT) was applied using standardised techniques and applicator templates. The BT dose was prescribed according to local practice. Pre-treatment orthogonal X-ray images verified the position of the BT applicator.

A detailed description of treatment techniques in relation to cancer diagnosis is provided in the Supplementary Appendix Table A1. For radiograph figures of typical treatment; see the Supplementary Appendix Figures A1–A4.

### Statistical analyses

The results from the questionnaire and the data from the medical records were coded and transferred to the freeware data-entry program Epi-Data (http://www.epidata.dk). We used Fisher's exact test (Monte Carlo approximation, 10^7^ samples) to test for differences in survivors and control characteristics (Table 1). The test was two-sided and at the 5% significance level.

Different cut-off levels were used to describe the frequency of symptom occurrence. For initial analyses, a symptom was dichotomised into having the symptom occasionally or more often and into not having the symptom at all the past 6 months. In the final analyses, the cut-off level was changed into at least once a week in symptoms that had a high prevalence, that is, exceeding 45% in controls. We calculated the proportions having each outcome (symptom) among cancer survivors and control women, and used relative risk (RR) defined as the ratio between these proportions as outcome measure. Age-adjusted RRs with 95% confidence intervals (CIs) were calculated according to the log-binomial model (McNutt *et al*, 2003) including age as a categorical covariate with the categorisation used in Table 1. Individuals with missing data were excluded from the calculations of each respective outcome. All calculations were performed using the SAS statistical software package (version 9.2, SAS Institute Inc., Cary, NC, USA).

## Results

In all, 616 of 789 (78%) cancer survivors and 344 of 478 (72%) control women returned a completed questionnaire and participated in the study (Figure 1). Median follow-up time was 74 months with a range of 26–179 months.

The median age for cancer survivors was 66.0 years and for control women 57.5 years (Table 1). Cancer survivors were more often single, had a lower level of education, lower degree of physical activity and were more often on disability pension compared with control women.

Nulliparity was twice as common among the survivors. Operational procedures at delivery and vaginal or perineal injury were less common among cancer survivors. The most common co-morbidity among cancer survivors was hypertension occurring in 38%, compared with 27% in control women. Diabetes mellitus and heart failure were also more prevalent among cancer survivors. Endometrial cancer and cervical cancer were the most common diagnoses. In all, 84% of the cancer survivors were treated for stage I–II disease.

**Figure 2:**
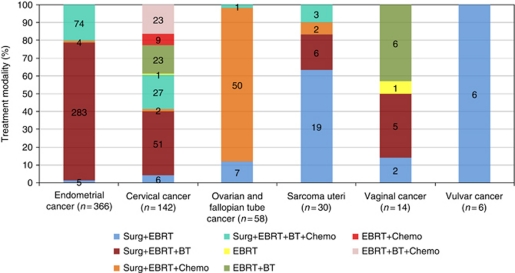
Treatment modality dependent on diagnosis. Abbreviations: Surg=surgery; EBRT=external beam radiation therapy; BT=brachytherapy; chemo=chemotherapy.

Overall 90% of the gynaecological cancer survivors were treated with surgery and EBRT with or without BT and chemotherapy. The remaining 10% consisted of a subset of cervical and vaginal cancer patients treated with radiation therapy alone (Figure 2). Additional information on demographic and clinical characteristics as well as a detailed description of treatment in relation to cancer diagnosis is provided in the Supplementary Appendix Table A1 and Supplementary Appendix Table A2. Treatment schedules for ovarian and fallopian tube cancer were identical and the results are hence presented together.

### Symptoms associated with cancer therapy

With a median follow-up time of 6.2 years, cancer survivors reported a higher occurrence of symptoms from the anal sphincter, the bowel, the urinary tract, symptoms related to sexuality, the pelvic bones and the lower abdomen and legs, compared with control women (Table 2 and Supplementary Appendix A3).

The highest age-adjusted RR among anal-sphincter symptoms was for ‘emptying of all stools into clothing without forewarning’, RR 12.7 (95% CI 4.0–40.3) with a prevalence of 12% among survivors and 0.9% among control women. This symptom was reported by one-third of the survivors with uterine sarcoma compared with 9% among survivors with endometrial and ovarian cancer. The prevalence of symptoms in relation to diagnosis is provided in the Supplementary Appendix Table A4. The occurrence of symptoms from the anal sphincter and bowel was in general higher among survivors with uterine sarcoma, cervical and vaginal cancer compared with endometrial and ovarian cancer survivors.

The highest age-adjusted RRs among urinary tract symptoms were observed for ‘difficulty feeling the need to empty the bladder’ (RR 2.8; 95% CI 1.5–5.4) with a prevalence of 9% in survivors compared with 3% in control women. The corresponding figures for ‘difficulty feeling a full bladder’ (RR 2.7; 95% CI 1.6–4.5), was 15% in cancer survivors compared with 5% in control women. These symptoms occurred more frequently among survivors treated with radiation therapy alone compared with those treated with surgery and radiation therapy.

For symptoms related to sexuality, 34% of the survivors reported ‘absence of vaginal elasticity’ compared with 14% among control women (RR 1.8; 95% CI 1.3–2.4). This symptom occurred in 47% of survivors treated with radiation therapy alone. ‘Deep dyspareunia when having intercourse’ occurred in 17% of cancer survivors irrespective of treatment modality, compared with 7% among control women (RR 3.7; 95% CI 2.4–5.7). About one-third of the survivors reported a ‘decreased ability for intercourse leading to a lower intercourse frequency’.

Pubic bone pain was at least three times as common among survivors compared with control women. The highest age-adjusted RR was for ‘pubic pain when walking indoors’, RR 4.9 (95% CI 2.1–11.6). Among survivors treated with radiation therapy alone, this symptom was reported by 18% compared with 2% among control women (RR 10.3; 95% CI 4.0–26.7).

For the abdomen and legs the highest age-adjusted RR was for ‘erysipelas on abdomen or legs’, RR 3.6 (95% CI 1.0–12.8) followed by ‘lower abdominal heaviness’, RR 2.1 (95% CI 1.5–3.0) occurring in one-fifth of the survivors. The prevalence among survivors treated with radiation therapy alone was 30%, compared with 11% among control women.

## Discussion

Gynaecological cancer survivors treated with pelvic radiation therapy alone or as part of combined treatment reported higher occurrence of late specific symptoms from all normal tissues addressed in the study, that is, the anal sphincter, the bowel, the urinary tract, the pelvic bones, the lower abdomen and legs as well as symptoms related to sexuality compared with matched control women.

Although gastrointestinal side-effects following pelvic radiation therapy are well documented, the occurrence and specificity of anal sphincter and bowel symptoms in long-term survivors have been less well studied (Andreyev, 2005; Putta and Andreyev, 2005). We have in a previous publication reported on our findings of higher occurrence of gastrointestinal symptoms and a 12-fold higher occurrence of emptying of all stools into clothing without forewarning among long-term gynaecological cancer survivors compared with controls. This severe faecal incontinence symptom was more prevalent in survivors treated for cervical cancer and uterine sarcoma. These diagnoses had in general higher median EBRT doses. In a previous study from our group, 65 prostate cancer survivors were asked by means of a study-specific postal questionnaire about bowel and urinary symptoms and sexual function 2–4 years after curative EBRT to the prostate. A correlation was found between the dose to the anal sphincter and faecal leakage in the interval of 45–55 Gy and also a correlation between defaecation urgency and loose stools and the dose to the rectum in the interval of 25–42 Gy (al-Abany *et al*, 2005). It is reasonable to also investigate the impact of dose to bowel and anal sphincter in relation to the occurrence of emptying of all stools without forewarning.

The urinary bladder has been regarded as less radiosensitive compared with the bowel (Marks *et al*, 1995). Late urinary side-effects appear to be less common but there are few published reports on the prevalence of specific urinary symptoms among long-term gynaecological cancer survivors. With median follow-up of 60 and 68 months, the PORTEC-1 trial reported a prevalence of 8% of mild genitourinary complications (measured by French-Italian glossary), while the GOG99 trial reported a 25% genitourinary complication rate (assessed by the 1985 GOG Adverse Events Criteria scale) after postoperative EBRT in women with early stages of endometrial cancer (Creutzberg *et al*, 2001; Keys *et al*, 2004). In the PORTEC-1 trial, the complications consisted of urinary urgency, recurrent urinary infections and minor incontinence while no details on specific symptoms are specified in the GOG 99 trial report. A higher risk of late urinary side-effects is reported for cervical cancer, which may be related to the higher doses of radiation therapy used (Elliott and Malaeb, 2011). In an observational study by Pieterse *et al* (2006), no increased occurrence of bladder dysfunction was reported at 24 months’ follow-up in 94 early-stage cervical cancer patients treated with postoperative EBRT, compared with surgery alone or control women. Nearly, every study shows that late post-radiation urinary morbidity continues to progress decades after radiation therapy. The short follow-up period and the restriction to only two questions concerning micturition may have contributed to the results reported by Pieterse *et al* (2006). In a population-based survey of 291 cervical cancer survivors with an average follow-up period of 6.6 years, self-reported symptoms of frequent micturition were reported by 42 and 45% following radiation therapy alone or postoperative EBRT (Korfage *et al*, 2009). Urinary leakage was reported by 19 and 26% and difficulties emptying the bladder in 6% and 11%, respectively. These results are in line with our findings. Besides difficulties emptying the bladder, urinary incontinence, urgency and cystitis, the survivors in our study also reported difficulties feeling a full bladder. Our results indicate that late side-effects from the urinary tract following radiation therapy are underestimated and underreported.

Most previous population-based surveys addressing the impact of radiation therapy on normal tissues have focused on sexuality in survivors after treatment of cervical cancer. In a population-based cohort study of long-term early stage cervical cancer, survivors reported more sexual dysfunction resulting in considerable distress, compared with control women (Bergmark *et al*, 1999). The impact of radiation therapy on sexual function has since then been confirmed in several publications (Jensen *et al*, 2003; Frumovitz *et al*, 2005; Park *et al*, 2007; Korfage *et al*, 2009). Some results support the positive effect of using vaginal dilators to prevent development of vaginal stenosis following radiation therapy as reviewed by the Cochrane Collaboration (Denton and Maher, 2003).

We have in a previous publication reported on the findings of higher occurrence of pubic bone symptoms and increased frequency of pubic bone pain with mean absorbed dose exceeding 52.5 Gy to the pubic bone (Waldenström *et al*, 2010). Cancer survivors in our study treated with radiation therapy alone also reported a higher prevalence of hip pain. The potential relationship between hip pain and radiation dose will be addressed in a coming report from our group.

Lymph oedema is a common late symptom among gynaecological cancer survivors (Bergmark *et al*, 2006). In a population-based mailed survey of 802 gynaecological cancer survivors (Beesley *et al*, 2007), 25% were diagnosed with lymph oedema or had symptomatic lower limb swelling. A higher prevalence was observed in vulvar cancer survivors. These results are consistent with our findings. The survivors in our study also report a higher occurrence of heaviness or swollen lower abdomen. The prevalence was higher after radiation therapy alone than after combination of surgery and radiation therapy. One may speculate that fibrosis or occlusion of lymph vessels after radiation therapy may be involved in the mechanism behind this symptom.

Some of the strengths of this study are the large population-based patient cohort and the high participation rate. Access to all medical records has ensured correct information regarding clinical characteristics and detailed knowledge of administered treatment. The construction of the study-specific questionnaire in close cooperation with the gynaecological cancer survivors, comprehensive face-to-face validation and the use of a privately answered questionnaire lowered the risk for measurement errors and eliminated interviewer-induced bias. By asking for symptom occurrence during the past 6 months, we avoided capturing temporary symptoms produced by chance and at the same time minimised risk of recall-induced problems. Symptoms were atomised, subdivided into very specific questions, which may have contributed to survivors’ multitude of symptoms. In an effort to increase participation, we did not include subjects over the age of 80 years. We cannot, however, exclude the possibility that non-participating subjects would have answered differently. The loss of information from part of the targeted-person-time may result in a deviation between the true effect measure and the observed effect measure in a way that is unpredictable. The minimum internal response rate for individual questions was 81% (502 out of 616) for survivors and 88% (302 out of 344) for control women in questions dealing with sexuality. This may negatively affect the generalisability of the study result. Individuals who failed to respond to a certain question were excluded from the analysis of that outcome. In all tables, the response rates can be assessed through the denominator that is presented for all items.

Research regarding long-term treatment effects in cancer survivors will give us knowledge for developing new strategies in cancer treatment. Updated recommendations are gradually introduced into clinical use. Several randomised clinical trials investigating the outcome of adjuvant radiation therapy in endometrial cancer patients have shown an increased risk of late radiation therapy-induced morbidity but without survival benefit (Creutzberg *et al*, 2001; Keys *et al*, 2004; Nout *et al*, 2009). Hence, adjuvant radiation therapy is nowadays seldom given to endometrial patients with low-intermediate risk malignancy. A potential new treatment approach is TGF-*β*, which is a promising target for preventing radiotherapy-induced fibrosis (Anscher, 2010). In addition, we also believe that prevention of some symptoms may be achieved by optimising radiation delivery by improved target imaging and treatment planning with dose-constraints guidelines and by the use of intensity modulated radiation therapy (Small *et al*, 2008).

In this first stage of our gynaecological cancer survivorship programme, we report on the occurrence of symptoms from normal tissues after pelvic irradiation. In the second stage of our programme, we will continue the reporting of the impact of specific symptoms on daily life activities and quality of life. The study cohort is highly heterogeneous and these circumstances contribute to variability of the absorbed dose of ionising radiation to the normal tissue surrounding the target, which will enable us to study the dose-volume effects of radiation therapy to normal-tissues in relation to self-reported symptoms. In the third stage of our survivorship programme, we intend to explore which levels of ionising radiation delivered to specific volumes of normal tissue surrounding the target that contribute to the occurrence of a specific symptom, which can affect quality of life. This may help the radiotherapist to optimise dose planning and thereby hopefully reduce the symptom burden among future long-term gynaecological cancer survivors.

## Figures and Tables

**Table 1:** Demographic characteristics for gynaecological cancer survivors and control women

	**Cancer survivors, *N*=616 (%)**	**Control women, *N*=344 (%)**	***P*-value** a
*Age*			<0.001
28–49	64/616 (10)	102/342 (30)	
50–59	102/616 (17)	80/342 (23)	
60–69	233/616 (38)	78/342 (23)	
70–79	217/616 (35)	82/342 (24)	
Median age, years (range)	66.0 (28–79)	57.5 (36–79)	
			
*Marital status*			0.009
Married or living with a partner	344/613 (56)	220/344 (64)	
Has a partner but lives alone	37/613 (6)	22/344 (6)	
Widow	84/613 (14)	37/344 (11)	
Single	148/613 (24)	65/344 (19)	
			
*Education*			<0.001
Elementary school	196/615 (32)	69/342 (20)	
Secondary school	236/615 (38)	146/342 (43)	
College or university	183/615 (30)	127/342 (37)	
			
*Employment*			<0.001
Student	5/613 (<1)	2/343 (<1)	
Unemployed	12/613 (2)	6/343 (2)	
Employed	202/613 (33)	188/343 (55)	
Housewife, other	11/613 (2)	5/343 (1)	
On sick leave	11/613 (2)	10/343 (3)	
Disability pension	53/613 (9)	15/343 (4)	
Retired	319/613 (52)	117/343 (34)	
			
*Country of birth*			<0.001
Sweden	510/614 (83)	316/344 (92)	
			
*Place of residency*			0.402
Rural district	52/615 (10)	34/341 (10)	
Village/town	191/615 (31)	93/341 (27)	
>500 000 citizen	372/615 (60)	214/341 (63)	
			
*Smoking*			0.608
Current smoker	144/607 (24)	88/343 (26)	
Former smoker	183/607 (30)	108/343 (31)	
Never smoker	280/607 (46)	147/343 (43)	
			
*BMI* b			0.256
<18.5 (underweight)	16/575 (3)	5/327 (2)	
18.5–25.0 (normal weight)	261/575 (45)	163/327 (50)	
25.0–30.0 (overweight)	201/575 (35)	116/327 (35)	
>30.0 (obese)	97/575 (17)	43/327 (13)	
BMI, median (range)	25.1 (16.0–53.1)	24.7 (13.4–44.5)	
			
*Exercise*			0.001
Never	76/599 (13)	20/341 (6)	
Occasionally – at least once a month	78/599 (13)	59/341 (17)	
At least once a week	445/599 (74)	262/341 (77)	
			
*Parity*			<0.001
Nulli (never given birth)	154/615 (25)	45/344 (13)	
1–3 para	410/615 (67)	280/344 (81)	
>3 para	51/615 (8)	19/344 (6)	
			
*Delivery*			
Fast <5 h	250/607 (41)	147/342 (43)	0.631
Slow >24 h	141/607 (23)	88/342 (26)	0.386
Vacuum	41/607 (7)	43/342 (13)	0.004
Forceps	12/607 (2)	7/342 (2)	1.000
Episiotomy	132/607 (22)	117/342 (34)	<0.001
Caesarean	28/607 (5)	40/342 (12)	0.001
Breech birth	18/607 (3)	20/342 (6)	0.038
			
*Delivery with birth weight >4 kg*			0.266
1	78/612 (13)	54/344 (16)	
⩾2	42/612 (7)	29/344 (8)	
			
*Pelvic floor injury*			
Vaginal or perineal injury	111/599 (19)	101/342 (30)	<0.001
Anal sphincter injury	18/593 (3)	18/342 (5)	0.111
			
*Intercurrent diseases*			
Previous abdominal surgery	264/616 (43)	156/344 (45)	0.456
Diabetes mellitus	58/611 (9)	17/338 (5)	0.016
Angina pectoris	32/600 (5)	11/341 (3)	0.147
Cardiac infarction	18/600 (3)	5/341 (1)	0.188
Heart failure	33/600 (6)	8/341 (2)	0.029
Hypertension	227/600 (38)	91/341 (27)	<0.001
Crohn's disease, treatment for	1/594 (<1)	0/332 (0)	1.000
Ulcerative colitis, treatment for	5/581 (<1)	7/327 (2)	0.131
Irritable bowel syndrome, treatment for	27/594 (4)	13/332 (4)	0.737
Haemorrhoids, treatment for	61/591 (10)	45/322 (14)	0.105
Lactose intolerance	33/598 (6)	13/331 (4)	0.334
Gluten intolerance	8/604 (1)	3/330 (<1)	0.755
Pelvic organ prolapse, treatment for	12/596 (2)	13/332 (4)	0.094
Rheumatism	37/600 (6)	19/341 (6)	0.775
Kidney disease	19/600 (3)	8/341 (2)	0.546
Lung disease	40/600 (7)	12/341 (4)	0.053
Thrombosis	47/600 (8)	16/341 (5)	0.077
Osteoporosis	58/600 (10)	25/341 (7)	0.235
Psychological disorders	78/600 (13)	43/341 (13)	0.919
Neurological disorders^c^	15/600 (3)	3/341 (<1)	0.089
			
*Medication*			
Using any kind of medication	439/606 (72)	194/338 (57)	<0.001
Oestrogen	224/606 (37)	50/339 (15)	<0.001

Abbreviation: BMI=body mass index.

Denominator is dependent on number of respondents answering a specific item and may differ from the maximum sum. Percentage may not total hundred because of rounding.

a *P*-value according to Fisher's exact test.

b Current BMI at the time the questionnaire was completed.

c Parkinson's disease, multiple sclerosis and epilepsy.

**Table 2:** Late symptoms during the past 6 months among gynaecological cancer survivors treated with pelvic radiation therapy with or without surgery and control women

	**Survivors, *N=*616 (%)**	**Controls, *N=*344 (%)**	**Survivors *vs* controls Age-adjusted RR (95% CI )**	**RT with surgery, *N=*549 (%)**	**RT with surgery *vs* controls Age-adjusted RR (95% CI)**	**RT without surgery, *N*=67 (%)**	**RT without surgery *vs* controls Age-adjusted RR (95% CI)**
*Anal-sphincter symptoms*							
Emptying of all stools into clothing without forewarning at least occasionally	70/606 (12)	3/344 (0.9)	**12.7 (4.0–40.3)**	55/546 (10)	**8.8 (2.8–28.3)**	15/60 (25)	**30.3 (9.1–101.1)**
Leakage of loose stools while awake at least occasionally	199/608 (33)	18/344 (5)	**6.0 (3.7–9.6)**	171/545 (31)	**5.5 (3.4–8.8)**	28/63 (44)	**8.5 (5.0–14.5)**
Leakage of loose stools while asleep at least occasionally	72/611 (12)	8/343 (2)	**5.5 (2.6–11.4)**	58/548 (11)	**4.4 (2.1–9.2)**	14/63 (22)	**9.8 (4.3–22.4)**
Anal leakage of mucus while asleep at least occasionally	32/607 (5)	4/344 (1)	**4.9 (1.7–14.1)**	25/545 (5)	**3.3 (1.1–9.4)**	7/62 (11)	**9.7 (2.9–32.3)**
Leakage of solid stools while awake at least occasionally	46/607 (8)	5/344 (1)	**4.4 (1.7–11.0)**	37/544 (7)	**3.4 (1.4–8.7)**	9/63 (14)	**9.3 (3.2–27.0)**
Faecal leakage without forewarning despite previous defaecation at least occasionally	188/605 (31)	23/344 (7)	**4.2 (2.8–6.4)**	168/545 (31)	**3.9 (2.6–6.0)**	20/60 (33)	**4.8 (2.8–8.2)**
Defaecation urgency with faecal leakage at least occasionally	298/603 (49)	42/343 (12)	**4.0 (3.0–5.4)**	262/541 (48)	**3.8 (2.8–5.2)**	36/62 (58)	**4.8 (3.3–6.8)**
Foul smelling flatulence at least once a week	116/602 (19)	22/343 (6)	**3.7 (2.4–5.8)**	102/541 (19)	**3.8 (2.4–5.9)**	14/61 (23)	**3.6 (2.0–6.7)**
Anal leakage of mucus while awake at least occasionally	87/603 (14)	14/343 (4)	**3.5 (2.0–6.1)**	76/542 (14)	**3.2 (1.8–5.6)**	11/61 (18)	**4.1 (1.9–8.6)**
Self-perception of faecal odour at least occasionally	108/606 (18)	18/340 (5)	**3.3 (2.0–5.4)**	95/544 (17)	**3.1 (1.9–5.2)**	13/62 (21)	**4.0 (2.1–7.8)**
Involuntary flatulence at least once a week	127/606 (21)	33/343 (10)	**2.4 (1.7–3.5)**	111/545 (20)	**2.3 (1.5–3.3)**	16/61 (26)	**2.8 (1.7–4.8)**
Unwanted defaecation while emptying bladder at least occasionally	238/603 (39)	57/342 (17)	**2.4 (1.9–3.2)**	213/541 (39)	**2.4 (1.8–3.1)**	25/62 (40)	**2.3 (1.6–3.4)**
Anal leakage of blood while awake at least occasionally	42/608 (7)	12/343 (4)	**2.0 (1.1–3.9)**	37/545 (7)	2.0 (1.0–3.9)	5/63 (8)	2.2 (0.8–6.7)
Involuntary loud flatulence at least occasionally	359/607 (59)	150/344 (44)	**1.4 (1.2–1.6)**	314/546 (58)	**1.3 (1.1–1.5)**	45/61 (74)	**1.7 (1.4–2.1)**
							
*Bowel symptoms*							
Defaecation urgency at least once a week	175/602 (29)	19/341 (6)	**5.7 (3.5–9.1)**	157/542 (29)	**5.7 (3.5–9.1)**	18/60 (30)	**6.0 (3.3–10.8)**
Protracted abdominal pain lasting >1 year, yes	69/593 (12)	15/339 (4)	**3.2 (1.9–5.6)**	59/532 (11)	**3.1 (1.7–5.5)**	10/61 (16)	**3.8 (1.8–7.9)**
Loose stools at least once a week	234/602 (39)	48/344 (14)	**3.0 (2.2–3.9)**	205/540 (38)	**2.9 (2.1–3.9)**	29/62 (47)	**3.5 (2.4–5.1)**
Abdominal pain and vomiting at least occasionally	60/605 (10)	13/344 (4)	**2.6 (1.4–4.7)**	49/545 (9)	**2.2 (1.2–4.1)**	11/60 (18)	**4.8 (2.2–10.1)**
Mucus in stools at least occasionally	156/607 (26)	45/343 (13)	**2.1 (1.5–3.0)**	143/545 (26)	**2.2 (1.6–3.0)**	13/62 (21)	1.6 (0.9–2.8)
Abdominal bloating at least once a week	147/605 (24)	60/342 (18)	**1.8 (1.4–2.3)**	129/544 (24)	**1.8 (1.3–2.3)**	18/61 (30)	**1.8 (1.1–2.7)**
Rectal bleeding at least occasionally	103/604 (17)	42/341(12)	**1.6 (1.1–2.2)**	92/545 (17)	**1.6 (1.2–2.4)**	11/59 (19)	1.5 (0.8–2.8)
Abdominal pain and stools at least occasionally	198/602 (33)	79/343 (23)	**1.6 (1.3–2.0)**	171/541 (32)	**1.5 (1.2–1.9)**	27/61 (44)	**2.0 (1.4–2.8)**
Abdominal pain at least occasionally	307/604 (51)	137/340 (40)	**1.4 (1.2–1.6)**	272/544 (50)	**1.4 (1.2–1.6)**	35/60 (58)	**1.5 (1.2–1.9)**
Abdominal pain and bloating at least occasionally	228/603 (38)	106/340 (31)	**1.4 (1.2–1.7)**	199/542 (37)	**1.4 (1.2–1.7)**	29/61 (48)	**1.6 (1.2–2.1)**
							
*Urinary tract symptoms*							
Difficulty feeling the need to empty bladder at least occasionally	56/604 (9)	11/343 (3)	**2.8 (1.5–5.4)**	46/544 (8)	**2.5 (1.3–5.0)**	10/60 (17)	**5.1 (2.3–11.3)**
Difficulty emptying bladder at least occasionally	49/602 (8)	11/343 (3)	**2.7 (1.4–5.2)**	43/542 (8)	**2.7 (1.4–5.5)**	6/60 (10)	**3.2 (1.3–8.4)**
Difficulty feeling full bladder at least occasionally	90/602 (15)	18/342 (5)	**2.7 (1.6–4.5)**	76/543 (14)	**2.4 (1.4–4.1)**	14/59 (24)	**4.5 (2.4–8.4)**
Haematuria at least occasionally	20/606 (3)	5/344 (1)	2.5 (0.9–6.9)	16/546 (3)	2.4 (0.8–6.8)	4/60 (7)	**5.0 (1.4–17.8)**
Straining to initiate emptying of bladder at least occasionally	77/605 (13)	21/343 (6)	**2.2 (1.4–3.7)**	68/545 (12)	**2.4 (1.5–3.9)**	9/60 (15)	**2.5 (1.2–5.3)**
Painful emptying of bladder at least occasionally	60/605 (10)	20/341 (6)	**2.0 (1.2–3.3)**	52/545 (10)	**1.9 (1.1–3.3)**	8/60 (13)	**2.3 (1.1–5.0)**
Urinary incontinence without urinary urgency at least occasionally	88/603 (15)	24/342 (7)	**1.8 (1.2–2.9)**	81/543 (15)	**1.9 (1.2–3.0)**	7/60 (12)	1.6 (0.7–3.6)
Night-time emptying of bladder at least twice per night or more, yes	222/607 (37)	58/341 (17)	**1.8 (1.4–2.4)**	199/548 (36)	**1.8 (1.4–2.4)**	23/59 (39)	**2.2 (1.5–3.2)**
Need of antibiotics because of urinary tract infection twice or more, yes	93/605 (15)	31/344 (9)	**1.6 (1.1–2.3)**	86/545 (16)	**1.7 (1.1–2.5)**	7/60 (12)	1.3 (0.6–2.9)
Urinary incontinence because of urinary urgency at least occasionally	209/607 (34)	66/342 (19)	**1.6 (1.2–2.0)**	186/545 (34)	**1.5 (1.2–2.0)**	23/62 (37)	**1.8 (1.2–2.7)**
Slow emptying of bladder at least occasionally	122/605 (20)	47/344 (14)	**1.6 (1.2–2.2)**	106/546 (19)	**1.5 (1.1–2.2)**	16/59 (27)	**2.0 (1.2–3.3)**
Self-perception of urine odour at least occasionally	149/605 (25)	62/341(18)	**1.4 (1.1–1.9)**	126/544 (23)	**1.3 (1.0–1.8)**	23/61 (38)	**2.1 (1.4–3.0)**
Urinary urgency at least occasionally	346/605 (57)	139/343 (41)	**1.3 (1.1–1.5)**	308/544 (57)	**1.3 (1.1–1.5)**	38/61 (62)	**1.5 (1.2–1.8)**
Feeling of incomplete bladder emptying at least occasionally	232/604 (38)	101/343 (29)	**1.3 (1.1–1.6)**	203/544 (37)	**1.3 (1.0–1.6)**	29/60 (48)	**1.6 (1.2–2.2)**
							
*Symptoms related to sexuality*							
Protracted genital pain lasting for >1 year, yes	28/593 (5)	4/339 (1)	**5.0 (1.7–14.5)**	21/532 (4)	**4.3 (1.4–13.0)**	7/61 (11)	**9.6 (2.9–31.8)**
Genital bleeding during or after intercourse at least once, yes	54/585 (9)	13/329 (4)	**3.7 (2.1–6.7)**	45/523 (9)	**3.6 (2.0–6.7)**	9/62 (15)	**3.9 (1.8–8.4)**
Deep dyspareunia when having intercourse, at least a little	101/583 (17)	23/330 (7)	**3.7 (2.4–5.7)**	90/521 (17)	**3.7 (2.4–5.8)**	11/62 (18)	**3.0 (1.6–5.7)**
Vaginal lubrication when sexually aroused, no	39/577 (7)	7/333 (2)	**2.9 (1.3–6.4)**	37/517 (7)	**2.9 (1.3–6.5)**	2/60 (3)	1.6 (0.3–7.5)
Decreased ability for intercourse leading to lower intercourse frequency, at least a little	176/575 (31)	37/327 (11)	**2.9 (2.1–4.1)**	160/515 (31)	**2.9 (2.0–4.0)**	11/60 (18)	**2.5 (1.5–4.1)**
Vaginal elasticity, no	172/502 (34)	41/302 (14)	**1.8 (1.3–2.4)**	148/451 (33)	**1.6 (1.2–2.2)**	24/51 (47)	**2.5 (1.7–3.7)**
Genital swelling when sexually aroused, no	82/571 (14)	35/327 (11)	**1.5 (1.0–2.2)**	72/512 (14)	1.5 (1.0–2.2)	10/59 (17)	1.6 (0.9–3.1)
Superficial dyspareunia when having intercourse, at least a little	140/584 (24)	67/330 (20)	**1.5 (1.2–2.0)**	125/523 (24)	**1.5 (1.1–2.0)**	15/61 (25)	1.4 (0.8–2.2)
Sensitivity to touch inside vagina, no	221/540 (41)	65/310 (21)	**1.3 (1.0–1.6)**	192/485 (40)	1.2 (1.0–1.5)	29/55 (53)	**1.7 (1.3–2.2)**
Sensitivity to touch of labia and clitoris, no	186/558 (33)	61/320 (19)	1.2 (0.9–1.5)	163/501 (33)	1.1 (0.9–1.4)	23/57 (40)	**1.5 (1.1–2.1)**
							
*Pelvic bone symptoms*							
Pubic pain when walking indoors at least occasionally	46/603 (8)	6/343 (2)	**4.9 (2.1–11.6)**	35/542 (6)	**4.1 (1.7–10.2)**	11/61 (18)	**10.3 (4.0–26.7)**
Pubic pain when walking outdoors 500 m at least occasionally	42/596 (7)	7/343 (2)	**3.7 (1.7–8.4)**	34/536 (6)	**3.3 (1.4–7.7)**	8/60 (13)	**6.6 (2.5–17.5)**
Pubic pain, yes	67/603 (11)	12/339 (4)	**3.4 (1.9–6.4)**	53/544 (10)	**3.0 (1.6–5.7)**	14/59 (24)	**7.0 (3.5–14.2)**
Hip pain, yes	212/599 (35)	113/343 (33)	1.0 (0.8–1.2)	182/538 (34)	0.9 (0.8–1.2)	30/61 (49)	**1.5 (1.1–2.0)**
Hip pain when walking outdoors 500 m at least occasionally	193/598 (32)	102/340 (30)	1.0 (0.8–1.3)	165/536 (31)	1.0 (0.8–1.2)	28/62 (45)	**1.5 (1.1–2.0)**
Sacral pain, yes	232/600 (39)	179/344 (52)	**0.8 (0.7–0.9)**	196/539 (36)	**0.7 (0.6–0.8)**	36/61 (59)	1.1 (0.9–1.4)
							
*Lower abdomen and leg symptoms*							
Erysipelas on abdomen or legs, yes	17/597 (3)	3/336 (1)	**3.6 (1.0–12.8)**	14/537 (3)	**4.0 (1.1–14.5)**	3/60 (5)	**5.2 (1.1–25.1)**
Lower abdominal heaviness at least occasionally	119/600 (20)	38/344 (11)	**2.1 (1.5–3.0)**	101/540 (19)	**2.1 (1.4–3.0)**	18/60 (30)	**2.7 (1.7–4.5)**
Pain in lower abdomen in connection with oedema at least occasionally	92/607 (15)	33/343 (10)	**1.9 (1.3–2.8)**	75/546 (14)	**1.7 (1.1–2.6)**	17/61 (28)	**3.0 (1.8–5.0)**
Leg pain in connection with oedema at least occasionally	164/606 (27)	54/342 (16)	**1.7 (1.3–2.3)**	145/545 (27)	**1.7 (1.2 (2.2)**	19/61 (31)	**2.0 (1.3–3.2)**
Swollen lower abdomen at least occasionally	121/599 (20)	51/342 (15)	**1.7 (1.3–2.4)**	106/538 (20)	**1.8 (1.3–2.5)**	15/61 (25)	**1.7 (1.0–2.8)**
Protracted leg pain lasting >1 year, yes	134/593 (23)	48/339 (14)	**1.5 (1.1–2.1)**	115/532 (22)	**1.4 (1.0–2.0)**	19/61 (31)	**2.2 (1.4–3.4)**
Swollen legs at least occasionally	218/606 (36)	94/344 (27)	**1.4 (1.1–1.7)**	197/546 (36)	**1.4 (1.1–1.9)**	21/60 (35)	1.4 (0.9–2.0)
Heavy legs at least occasionally	210/606 (35)	97/344 (28)	**1.1 (1.1–1.6)**	186/546 (34)	**1.3 (1.0–1.6)**	24/60 (40)	**1.4 (1.0–2.1)**

Abbreviations: CI=confidence interval; RR=relative risk; RT=radiation therapy.

Bold entries indicate statistic significance. The symptoms are sorted into anatomical region of supposed origin and in size order for RR, the number in the denominator may vary because of missing information.

## Supplementary Material

Supplementary Appendix Figure A1

Supplementary Appendix Figure A2

Supplementary Appendix Figure A3

Supplementary Appendix Figure A4

Supplementary Appendix Table A1

Supplementary Appendix Table A2

Supplementary Appendix Table A3

Supplementary Appendix Table A4
