# Supplementary material for: Late symptoms in long-term gynaecological cancer survivors after radiation therapy: a population-based cohort study
Source: Br J Cancer. 2011 Aug 16;105(6):737–45. doi: 10.1038/bjc.2011.315 (PMC3171018; doi:10.1038/bjc.2011.315)
Supplement: Supplementary Appendix Table A1 [file bjc2011315x5.pdf]

Appendix Table A1. Treatment Characteristics for Gynaecological Cancer Survivors in Relation to Diagnosis

	**Endometrial Cancer**	**Cervical Cancer**		**Ovarian and Fallopian Tube Cancer**	**Sarcoma Uteri**	**Vaginal Cancer**		**Vulvar Cancer**
		No surgery	***Surgery***			***No surgery***	***Surgery***	
	N=366 (%)	*N=56 (%)*	*N=86 (%)*	*N=58 (%)*	*N=30 (%)*	*N=7 (%)*	*N=7 (%)*	*N=6 (%)*
Surgery	366/366 (100)	0/56 (0)	86/86 (100)	58/58 (100)	30/30 (100)	0/7 (0)	7/7 (100)	6/6 (100)
TAH+/-SOE+/-omentectomy	338/366 (92)	N.A.	13/86 (15)	57/58 (98)	28/30 (93)	N.A	3/7 (43)	0/6 (0)
TAH+/-SOE+/-omentectomy+lymph node sampling	28/366 (8)	N.A.	0/86 (0)	1/58 (2)	2/30 (7)	N.A.	0/7 (0)	0/6 (0)
Radical hysterectomy+pelvic lymphadenectomy	0/366 (0)	N.A.	71/86 (83)	0/58 (0)	0/30 (0)	N.A	0/7 (0)	0/6 (0)
Vulvar resection+/-lymph node resection	0/366 (0)	N.A.	0/86 (0)	0/58 (0)	0/30 (0)	N.A		6/6 (100)
Other	0/366 (0)	N.A.	2/86 (2)	0/58 (0)	0/30 (0)	N.A	4/7 (57)	0/6 (0)
Brachytherapy	357/366 (98)	46/56 (82)	78/86 (91)	1/58 (2)	9/30 (30)	6/7 (86)	5/7 (71)	0/6 (0)
Isotope								
Radium+/-Cesium	25/366 (7)	0/56 (0)	2/86 (2)	0/58 (0)	1/30 (3)	0/7 (0)	0/7 (0)	0/6 (0)
Cesium	10/366 (3)	33/56 (59)	34/87 (40)	0/58 (0)	1/30 (3)	2/7 (29)	0/7 (0)	0/6 (0)
Iridium	322/366 (88)	13/56 (23)	42/87 (49)	1/58 (2)	7/30 (23)	4/7 (57)	5/7 (71)	0/6 (0)
Time relation to surgery								
Preoperative	29/366 (8)	0/56 (0)	29/86 (34)	0/58 (0)	1/30 (3)	N.A.	0/7 (0)	0/6 (0)
No surgery	0/366 (0)	46/56 (82)	0/86 (0)	0/58 (0)	0/30 (0)	6/7 (86)	N.A.	0/6 (0)
Postoperative	328/366 (90)	0/56 (0)	49/86 (57)	1/58 (2)	8/30 (27)	N.A.	5/7 (71)	0/6 (0)
Chemotherapy	78/366 (21)	32/56 (57)	29/86 (34)	51/58 (88)	5/30 (17)	0/7 (0)	0/7 (0)	0/6 (0)
Prior +/- after EBRT	78/366 (21)	15/56 (27)	14/86 (16)	51/58 (88)	5/30 (17)	0/7 (0)	0/7 (0)	0/6 (0)
Concomitant with EBRT	0/366 (0)	17/56 (30)	15/86 (17)	0/58 (0)	0/30 (0)	0/7 (0)	0/7 (0)	0/6 (0)

Appendix Table A1. Treatment Characteristics for Gynaecological Cancer Survivors in Relation to Diagnosis

	**Endometrial Cancer**	**Cervical Cancer**		**Ovarian and Fallopian Tube Cancer**	**Sarcoma Uteri**	**Vaginal Cancer**		**Vulvar Cancer**
		No surgery	***Surgery***			***No surgery***	***Surgery***	
	N=366 (%)	*N=56 (%)*	*N=86 (%)*	*N=58 (%)*	*N=30 (%)*	*N=7 (%)*	*N=7 (%)*	*N=6 (%)*
EBRT								
Dose (Gy)								
Total median dose, Gy (range)	40.0 (10.8-46.8)	55.0 (39.6-70.0)	45.0 (14.4-67.0)	40.0 (30.0-60.0)	49.9 (39.6-52.2)	50.4 (39.6-59.4)	43.2 (39.6-46.0)	43.6 (35.2-48.6)
< 40.0 Gy	146/366 (40)	1/56 (2)	10/86 (12)	6/58 (10)	1/30 (3)	2/7 (29)	3/7 (43)	2/6 (33)
40.0-44.9 Gy	79/366 (22)	2/56 (4)	24/86 (28)	51/58 (88)	6/30 (20)	0/7 (0)	0/7 (0)	2/6 (33)
45.0-55.0 Gy	141/366 (39)	32/56 (57)	51/86 (59)	1/58 (2)	23/30 (77)	4/7 (57)	4/7 (57)	2/6 (33)
> 55.0 Gy	0/366 (0)	20/56 (36)	1/86 (1)	0/58 (0)	0/30 (0)	1/7 (14)	0/7 (0)	0/6 (0)
Energy, median MV (range)	16 (6-50)	18 (6-50)	18(6-50)	18 (6-21)	18 (6-50)	18 (18-50)	18 (15-50)	18 (6-18)
Field technique								
Two opposing fields	68/365 (19)	1/55 (2)	15/86 (17)	48/58 (83)	7/30 (23)	1/7 (14)	0/7 (0)	2/6 (33)
Four-field box+/-regional lymph nodes	297/365 (81)	54/55 (98)	71/86 (83)	10/58 (17)	23/30 (77)	6/7 (86)	7/7 (100)	4/6 (67)
Target area								
Pelvic field	336/366 (92)	52/56 (93)	50/86 (58)	0/58 (0)	27/30 (90)	5/7 (71)	4/7 (57)	1/6 (17)
Abdominal field	19/366 (5)	0/56 (0)	1/86 (1)	58/58 (100)	2/30 (7)	0/7 (0)	0/13 (0)	0/6 (0)
Pelvic field+paraaortic lymph nodes	10/366 (3)	2/56 (4)	34/86 (40)	0/58 (0)	1/30 (3)	0/7 (0)	0/13 (0)	0/6 (0)
Pelvic or vulvar field+inguinal lymph nodes	1/366 (< 1)	2/56 (4)	1/86 (1)	0/58 (0)	0/30 (0)	2/7 (29)	8/13 (62)	5/6 (83)
Median time since radiation therapy, months (range)	69 (26-177)	66 (28-165)	77 (27-176)	115 (32-179)	81 (28-154)	53 (29-127)	60 (28-94)	105 (52-115)
Percentile 25th-75th	47-107	41-105	45-176	68-165	53-126	39-111	45-83	59-112

Abbreviations: TAH = total abdominal hysterectomy; SOE = salpingo-oophorectomy; EBRT = external beam radiation therapy; Gy = Gray; MV = megavoltage

Footnote:
Prescribed radiation treatment: Preoperative BT for **endometrial cancer** was practiced until 1994 (Kottmeier, 1964). It consisted of two manually deposited intrauterine insertions of ^{226}Radium implants and one intravaginal BT treatment of ^{137}Cesium covering the proximal two-thirds of the vagina. The ^{137}Cesium was positioned by an

afterloading system and the prescribed dose was 13 Gy. In 1995 primary surgery followed by EBRT and intravaginal BT using an afterloading device with ^{192}Iridium became standard. The prescribed postoperative BT dose was 5 Gy twice one week apart or 3.75 Gy three times one week apart. The prescribed EBRT dose was 38 at 2 Gy per fraction, 39.8 Gy at 1.8 Gy per fraction or 46 Gy at 2 Gy per fraction.

Preoperative BT for **early-stage cervical cancer**, FIGO IB-IIA, (Benedet *et al*, 2000) consisted of two uterovaginal insertions of ^{137}Cesium three weeks apart followed by either surgery or EBRT four weeks later. The total dose prescribed to Point A was 45 Gy in squamous cell carcinoma and 48 Gy in adenocarcinoma tumours. In women with primary surgery postoperative BT with ^{192}Iridium was administered at 5 Gy per fraction twice or at 4 Gy per fraction in three fractions. The prescribed EBRT dose was 45 Gy at 1.6 Gy per fraction or 46 Gy at 2 Gy per fraction. Until 2001 an additional prophylactic paraaortic treatment to 40 Gy at 1.6 Gy per fraction was given to patients with pelvic lymph node metastases. Patients treated with preoperative BT received EBRT with a four cm wide central shielding and the prescribed dose to the shielded volume was adjusted in order not to exceed a total dose of 50 Gy to the rectum and 60 Gy to the urinary bladder. From 2001 concomitant chemoradiation with weekly cisplatinum (40 mg/m^2) was introduced.

For **locally advanced stages of cervical cancer** the prescribed EBRT dose was 50 to 55 Gy in patients with BT and 60 to 67 Gy without BT. Prescribed BT dose was 10 Gy per fraction weekly up to three times or 4 Gy per fraction weekly three times. Neoadjuvant platinum-based combination chemotherapy was given prior to EBRT in some patients. Concomitant chemoradiation with weekly cisplatinum was gradually introduced in 1999.

The prescribed EBRT dose for **ovarian and fallopian tube cancer** was 20 Gy to the whole abdomen and an additional 20 Gy to an abdominal volume with lowered cranial margins corresponding to L2-L3, given at 1.6 Gy per fraction with opposing fields.

References:

Benedet JL, Bender H, Jones H, 3rd, Ngan HY, Pecorelli S (2000) FIGO staging classifications and clinical practice guidelines in the management of gynecologic cancers. FIGO Committee on Gynecologic Oncology. Int J Gynaecol Obstet 70: 209-62

Kottmeier HL (1964) Surgical and Radiation Treatment of Carcinoma of the Uterine Cervix. Experience by the Current Individualized Stockholm Technique. Acta Obstet Gynecol Scand 43: SUPPL2:1-48
