# Supplementary material for: Late symptoms in long-term gynaecological cancer survivors after radiation therapy: a population-based cohort study
Source: Br J Cancer. 2011 Aug 16;105(6):737–45. doi: 10.1038/bjc.2011.315 (PMC3171018; doi:10.1038/bjc.2011.315)
Supplement: Supplementary Appendix Table A2 [file bjc2011315x6.pdf]

Appendix Table A2. Demographic and Clinical Characteristics for Gynaecological Cancer Survivors in Relation to Diagnosis

	**Endometrial Cancer**	**Cervical Cancer**	**Ovarian and Fallopian Tube Cancer**	**Sarcoma Uteri**	**Vaginal Cancer**	**Vulvar Cancer**	**p-value [a]**
	N=366	*N=142*	*N=58*	*N=30*	*N=14*	*N=6*	
Age							<0.001
28-49	5/366 (1)	48/142 (34)	4/58 (7)	4/30 (13)	3/14 (21)	0/6 (0)	
50-59	39/366 (11)	40/142 (28)	14/58 (24)	3/30 (10)	6/14 (43)	0/6 (0)	
60-69	158/366 (43)	25/142 (18)	29/58 (50)	13/30 (43)	3/14 (21)	5/6 (83)	
70-79	164/366 (45)	29/142 (20)	11/58 (19)	10/30 (33)	2/14 (14)	1/6 (17)	
Median age, years (range)	69 (38-79)	55 (30-79)	64 (33-79)	66 (28-77)	58 (42-78)	65 (61-73)	
Marital status							0.680
Married or living with a partner	202/365 (55)	77/141 (55)	34/58 (59)	20/30 (67)	7/13 (54)	4/6 (67)	
Has a partner but lives alone	22/365 (6)	8/141 (6)	5/58 (9)	2/30 (7)	0/13 (0)	0/6 (0)	
Widow	58/365 (16)	16/141 (11)	5/58 (9)	2/30 (7)	1/13 (8)	2/6 (33)	
Single	83/365 (23)	40/141 (28)	14/58 (24)	6/30 (20)	5/13 (38)	0/6 (0)	
Education							0.628
Elementary school	114/365 (31)	43/142 (30)	19/58 (33)	14/30 (47)	4/14 (29)	2/6 (33)	
Secondary school	144/365 (40)	56/142 (39)	18/58 (31)	9/30 (30)	5/14 (36)	4/6 (67)	
College or university	107/365 (29)	43/142 (30)	21/58 (36)	7/30 (23)	5/14 (36)	0/6 (0)	
Employment							<0.001
Student	0/366 (0)	5/139 (4)	0/58 (0)	0/30 (0)	0/14 (0)	0/6 (0)	
Unemployed	5/366 (1)	3/139 (2)	1/58 (2)	1/30 (3)	1/14 (7)	1/6 (17)	
Employed	88/366 (24)	73/139 (53)	23/58 (40)	8/30 (27)	9/14 (64)	1/6 (17)	
Housewife, other	10/366 (3)	0/139 (0)	1/58 (2)	0/30 (0)	0/14 (0)	0/6 (0)	
On sick leave	2/366 (< 1)	6/139 (4)	2/58 (3)	1/30 (3)	0/14 (0)	0/6 (0)	
Disability pension	28/366 (8)	14/139 (10)	5/58 (9)	2/30 (7)	1/14 (7)	3/6 (50)	
Retired	233/366 (64)	38/139 (27)	26/58 (45)	18/30 (60)	3/14 (21)	1/6 (17)	
Country of birth							0.704
Sweden	307/366 (84)	113/141 (80)	48/58 (83)	26/30 (87)	12/14 (86)	4/6 (67)	
Place of residency							0.107
Rural district	29/366 (8)	12/142 (8)	7/58 (12)	3/29 (10)	1/14 (7)	0/6 (0)	
Village/town	124/366 (34)	34/142 (24)	11/58 (19)	13/29 (45)	6/14 (43)	3/6 (50)	
> 500 000 citizen	213/366 (58)	96/142 (68)	40/58 (69)	13/29 (45)	7/14 (50)	3/6 (50)	

Appendix Table A2. Demographic and Clinical Characteristics for Gynaecological Cancer Survivors in Relation to Diagnosis

	**Endometrial Cancer**	**Cervical Cancer**	**Ovarian and Fallopian Tube Cancer**	**Sarcoma Uteri**	**Vaginal Cancer**	**Vulvar Cancer**	**p-value [a]**
	N=366	*N=142*	*N=58*	*N=30*	*N=14*	*N=6*	
Smoking							<0.001
Current smoker	66/362 (18)	51/139 (37)	11/57 (19)	7/29 (24)	6/14 (43)	3/6 (50)	
Former smoker	105/362 (29)	40/139 (29)	25/57 (44)	6/29 (21)	5/14 (36)	2/6 (33)	
Never smoker	191/362 (53)	48/139 (35)	21//57 (37)	16/29 (55)	3/14 (21)	1/6 (17)	
BMI [b]							0.032
< 18.5 (underweight)	12/337 (4)	3/136 (2)	1/58 (2)	0/27 (0)	0/12 (0)	0/5 (0)	
18.5-25.0 (normal weight)	129/337 (38)	74/136 (54)	35/58 (60)	12/27 (44)	7/12 (58)	4/5 (80)	
25.0-30.0 (overweight)	131/337 (39)	38/136 (28)	19/58 (33)	10/27 (37)	3/12 (25)	0/5 (0)	
> 30.0 (obese)	65/337 (19)	21/136 (15)	3/58 (5)	5/27 (19)	2/12 (17)	1/5 (20)	
BMI, median (range)	26 (16-53)	24 (17-41)	24 (18-34)	25 (20-39)	24 (20-31)	24 (21-38)	
Exercise							0.117
Never	44/358 (12)	25/136 (18)	3/56 (5)	0/29 (0)	3/14 (21)	1/6 (17)	
Occasionally-at least once a month	45/358 (13)	18/136 (13)	9/56 (16)	4/29 (14)	1/14 (7)	1/6 (17)	
At least once a week	269/358 (75)	93/136 (68)	44/56 (79)	25/29 (86)	10/14 (71)	4/6 (67)	
Parity							0.005
Nulli (never given birth)	98/366 (27)	33/141 (23)	16/58 (28)	2/30 (7)	4/14 (29)	1/6 (17)	
1-3 para	246/366 (67)	84/141 (60)	40/58 (69)	25/30 (83)	10/14 (71)	5/6 (83)	
> 3 para	22/366 (6)	24/141 (17)	2/58 (3)	3/30 (10)	0/14 (0)	0/6 (0)	
Delivery							
Fast < 5 hours	144/359 (40)	61/140 (44)	23/58 (40)	15/30 (50)	6/14 (43)	1/6 (17)	0.722
Slow > 24 hours	79/359 (22)	33/140 (24)	14/58 (24)	10/30 (33)	2/14 (14)	3/6 (50)	0.395
Vacuum	20/359 (6)	9/140 (6)	8/58 (14)	2/30 (7)	1/14 (7)	1/6 (17)	0.182
Forceps	4/359 (1)	5/140 (4)	1/58 (2)	0/30 (0)	2/14 (14)	0/6 (0)	0.043
Episiotomy	73/359 (20)	32/140 (23)	17/58 (29)	7/30 (23)	2/14 (14)	1/6 (17)	0.702
Caesarean	15/359 (4)	7/140 (5)	2/58 (3)	2/30 (7)	2/14 (14)	0/6 (0)	0.469
Breech birth	9/359 (3)	6/140 (4)	0/58 (0)	1/30 (3)	1/14 (7)	1/6 (17)	0.109
Delivery with birth weight > 4 kg							0.931
1	51/364 (14)	16/141 (11)	7/58 (12)	2/29 (7)	2/14 (14)	0/6 (0)	
≥ 2	26/364 (7)	11/141 (8)	2/58 (3)	3/29 (10)	0/14 (0)	0/6 (0)	

Appendix Table A2. Demographic and Clinical Characteristics for Gynaecological Cancer Survivors in Relation to Diagnosis

	**Endometrial Cancer**	**Cervical Cancer**	**Ovarian and Fallopian Tube Cancer**	**Sarcoma Uteri**	**Vaginal Cancer**	**Vulvar Cancer**	**p-value [a]**
	N=366	*N=142*	*N=58*	*N=30*	*N=14*	*N=6*	
Pelvic floor injury							
Vaginal or perineal injury	63/354 (18)	26/138 (19)	9/58 (16)	9/29 (31)	2/14 (14)	2/6 (33)	0.440
Anal sphincter injury	11/351 (3)	2/137 (2)	2/57 (4)	2/29 (7)	1/13 (8)	0/6 (0)	0.337
Intercurrent diseases							
Previous abdominal surgery	175/366 (48)	44/142 (31)	26/58 (45)	10/30 (33)	5/14 (36)	4/6 (7)	0.009
Diabetes mellitus	40/365 (11)	6/139 (5)	6/58 (10)	5/29 (17)	0/14 (0)	1/6 (17)	0.061
Angina pectoris	19/361 (5)	6/134 (2)	2/57 (4)	2/29 (7)	2/13 (15)	1/6 (17)	0.272
Cardiac infarction	15/361 (4)	2/134 (1)	0/57 (0)	0/29 (0)	1/13 (8)	0/6 (0)	0.265
Heart failure	23/361 (6)	4/134 (3)	4/57 (7)	1/29 (3)	0/13 (0)	1/6 (17)	0.375
Hypertension	164/361 (45)	31/134 (23)	19/57 (33)	8/29 (28)	3/13 (23)	2/6 (33)	<0.001
Crohn’s disease, treatment for	1/353 (< 1)	0/136 (0)	0/57 (0)	0/29 (0)	0/14 (0)	0/5 (0)	1.000
Ulcerative colitis, treatment for	2/343 (< 1)	3/135 (2)	0/57 (0)	0/27 (0)	0/14 (0)	0/5 (0)	0.422
Irritable bowel syndrome, treatment for	12/352 (3)	10/136 (7)	5/58 (9)	0/28 (0)	0/14 (0)	0/6 (0)	0.200
Hemorrhoids, treatment for	42/351 (12)	8/134 (6)	7/58 (12)	0/29 (0)	4/14 (29)	0/5 (0)	0.024
Lactose intolerance	16 /357 (4)	8/134 (6)	1/58 (2)	2/29 (7)	4/14 (29)	2/6 (33)	0.002
Gluten intolerance	3/360 (< 1)	4/137 (3)	0/58 (0)	0/29 (0)	1/14 (7)	0/6 (0)	0.149
Pelvic organ prolapse, treatment for	9/355 (3)	1/136 (< 1)	1/57 (2)	1/29 (3)	0/14 (0)	0/5 (0)	0.610
Rheumatism	26/361 (7)	5/134 (4)	5/57 (9)	1/29 (3)	0/13 (0)	0/6 (0)	0.622
Kidney disease	10/361 (3)	6/134 (4)	1/57 (2)	1/29 (3)	1/13 (8)	0/6 (0)	0.573
Lung disease	27/361 (7)	6/134 (4)	4/57 (7)	1/29 (3)	0/13 (0)	2/6 (33)	0.183
Thrombosis	29/361 (8)	10/134 (7)	5/57 (9)	1/29 (3)	1/13 (8)	1/6 (17)	0.810
Osteoporosis	36/361 (10)	9/134 (7)	7/57 (12)	5/29 (17)	0/13 (0)	1/6 (17)	0.295
Psychological disorders	49/361 (14)	15/134 (11)	6/57 (11)	4/29 (14)	1/13 (8)	3/6 (50)	0.229
Neurological disorders [c]	15/361 (4)	0/134 (0)	0/57 (0)	0/29 (0)	0/13 (0)	0/6 (0)	0.079
Medication							
Using any kind of medication	273/362 (75)	93/137 (68)	43/58 (74)	19/29 (66)	8/14 (57)	3/6 (50)	0.185
Oestrogen	103/359 (29)	85/140 (61)	24/58 (41)	6/30 (20)	5/13 (38)	1/6 (17)	<0.001

Appendix Table A2. Demographic and Clinical Characteristics for Gynaecological Cancer Survivors in Relation to Diagnosis

	**Endometrial Cancer** *N=366*	**Cervical Cancer** *N=142*	**Ovarian and Fallopian Tube Cancer** *N=58*	**Sarcoma Uteri** *N=30*	**Vaginal Cancer** *N=14*	**Vulvar Cancer** *N=6*	**p-value** [a]
Stage of malignancy							<0.001
Stage I	258/366 (70)	64/142 (45)	25/58 (43)	20/30 (67)	10/14 (71)	0/6 (0)	
Stage II	61/366 (17)	48/142 (34)	23/58 (40)	4/30 (13)	4/14 (29)	1/6 (17)	
Stage III	46/366 (13)	20/142 (14)	10/58 (17)	3/30 (10)	0/14 (0)	5/6 (83)	
Stage IV	1/366 (< 1)	1/142 (< 1)	0/58 (0)	1/30 (3)	0/14 (0)	0/6 (0)	
Stage unknown	0/366 (0)	9/142 (6)	0/58 (0)	2/30 (7)	0/14 (0)	0/6 (0)	
Summary of treatment							<0.001
Surgery and External Radiation Therapy, Total	366/366 (100)	86/142 (61)	58/58 (100)	30/30 (100)	7/14 (50)	6/6 (100)	
Surgery and External radiation therapy alone	5/366 (1)	6/142 (4)	7/58 (12)	19/30 (63)	2/14 (14)	6/6 (100)	
& Brachytherapy	283/366 (77)	51/142 (36)	0/58 (0)	6/30 (20)	5/14 (36)	0/6 (0)	
& Chemotherapy	4/366 (1)	2/142 (1)	50/58 (86)	2/30 (7)	0/14 (0)	0/6 (0)	
& Brachytherapy + Chemotherapy	74/366 (20)	27/142 (19)	1/58 (2)	3/30 (10)	0/14 (0)	0/6 (0)	
No surgery External Radiation Therapy, Total	0/366 (0)	56/142 (39)	0/58 (0)	0/30 (0)	7/14 (50)	0/6 (0)	
External radiation therapy alone	0/366 (0)	1/142 (< 1)	0/58 (0)	0/30 (0)	1/14 (7)	0/6 (0)	
& Brachytherapy	0/366 (0)	23/142 (16)	0/58 (0)	0/30 (0)	6/14 (43)	0/6 (0)	
& Chemotherapy	0/366 (0)	9/142 (6)	0/58 (0)	0/30 (0)	0/14 (0)	0/6 (0)	
& Brachytherapy + Chemotherapy	0/366 (0)	23/142 (16)	0/58 (0)	0/30 (0)	0/14 (0)	0/6 (0)	
Median time since radiation therapy, months (range)	69 (26-177)	74 (27-176)	115 (32-179)	81 (28-154)	56 (27-127)	105 (52-115)	
Percentile 25th-75th	47-107	44-112	68-165	53-126	45-94	59-112	

Abbreviations: BMI = body mass index

[a] *p*-value according to Fisher's exact test, [b] current body mass index at the time the questionnaire was completed, [c] Parkinson's disease, multiple sclerosis and epilepsy

Denominator is dependent on number of respondents answering a specific item and may differ from the maximum sum, percentage may not total hundred because of rounding
