# Supplementary material for: Late symptoms in long-term gynaecological cancer survivors after radiation therapy: a population-based cohort study
Source: Br J Cancer. 2011 Aug 16;105(6):737–45. doi: 10.1038/bjc.2011.315 (PMC3171018; doi:10.1038/bjc.2011.315)
Supplement: Supplementary Appendix Table A3 [file bjc2011315x7.pdf]

Appendix Table A3. Late Symptoms during the Past Six Months among Gynaecological Cancer Survivors Treated with Pelvic Radiation Therapy with or without Surgery and Control Women, all symptoms

	**Survivors** *N=616 (%)*	**Controls** *N=344 (%)*	**Survivors vs. Controls Age adjusted RR (95% CI)**	**RT with Surgery** *N=549 (%)*	**RT with Surgery vs. Controls Age adjusted RR (95% CI)**	**RT without Surgery** *N= 67 (%)*	**RT without Surgery vs. Controls Age adjusted RR (95% CI)**
Anal-sphincter symptoms							
Emptying of all stools into clothing without forewarning at least occasionally	70/606 (12)	3/344 (0.9)	**12.7 (4.0-40.3)**	55/546 (10)	**8.8 (2.8-28.3)**	15/60 (25)	**30.3 (9.1-101.1)**
Leakage of loose stools while awake at least occasionally	199/608 (33)	18/344 (5)	**6.0 (3.7-9.6)**	171/545 (31)	**5.5 (3.4-8.8)**	28/63 (44)	**8.5 (5.0-14.5)**
Leakage of loose stools while asleep at least occasionally	72/611 (12)	8/343 (2)	**5.5 (2.6-11.4)**	58/548 (11)	**4.4 (2.1-9.2)**	14/63 (22)	**9.8 (4.3-22.4)**
Anal leakage of mucus while asleep at least occasionally	32/607 (5)	4/344 (1)	**4.9 (1.7-14.1)**	25/545 (5)	**3.3 (1.1-9.4)**	7/62 (11)	**9.7 (2.9-32.3)**
Leakage of solid stools while awake at least occasionally	46/607 (8)	5/344 (1)	**4.4 (1.7-11.0)**	37/544 (7)	**3.4 (1.4-8.7)**	9/63 (14)	**9.3 (3.2-27.0)**
Faecal leakage without forewarning despite previous defaecation at least occasionally	188/605 (31)	23/344 (7)	**4.2 (2.8-6.4)**	168/545 (31)	**3.9 (2.6-6.0)**	20/60 (33)	**4.8 (2.8-8.2)**
Defaecation urgency with fecal leakage at least occasionally	298/603 (49)	42/343 (12)	**4.0 (3.0-5.4)**	262/541 (48)	**3.8 (2.8-5.2)**	36/62 (58)	**4.8 (3.3-6.8)**
Foul smelling flatulence at least once a week	116/602 (19)	22/343 (6)	**3.7 (2.4-5.8)**	102/541 (19)	**3.8 (2.4-5.9)**	14/61 (23)	**3.6 (2.0-6.7)**
Anal leakage of mucus while awake at least occasionally	87/603 (14)	14/343 (4)	**3.5 (2.0-6.1)**	76/542 (14)	**3.2 (1.8-5.6)**	11/61 (18)	**4.1 (1.9-8.6)**
Self-perception of faecal odor at least occasionally	108/606 (18)	18/340 (5)	**3.3 (2.0-5.4)**	95/544 (17)	**3.1 (1.9-5.2)**	13/62 (21)	**4.0 (2.1-7.8)**
Anal leakage of blood while asleep at least occasionally	9/608 (1)	2/343 (0.5)	2.7 (0.6-12.7)	8/546 (1)	2.7 (0.5-13.1)	1/62 (2)	2.9 (0.3-29.4)
Involuntary flatulence at least once a week	127/606 (21)	33/343 (10)	**2.4 (1.7-3.5)**	111/545 (20)	**2.3 (1.5-3.3)**	16/61 (26)	**2.8 (1.7-4.8)**
Unwanted defaecation while emptying bladder at least occasionally	238/603 (39)	57/342 (17)	**2.4 (1.9-3.2)**	213/541 (39)	**2.4 (1.8-3.1)**	25/62 (40)	**2.3 (1.6-3.4)**
Anal leakage of blood while awake at least occasionally	42/608 (7)	12/343 (4)	**2.0 (1.1-3.9)**	37/545 (7)	2.0 (1.0-3.9)	5/63 (8)	2.2 (0.8-6.7)
Involuntary loud flatulence at least occasionally	359/607 (59)	150/344 (44)	**1.4 (1.2-1.6)**	314/546 (58)	**1.3 (1.1-1.5)**	45/61 (74)	**1.7 (1.4-2.1)**
Anal pain at least occasionally	129/608 (21)	64/343 (19)	1.2 (0.9-1.6)	116/548 (21)	1.2 (0.9-1.6)	13/60 (22)	1.2 (0.7-2.0)
Pruritus ani at least occasionally	200/609 (33)	114/343 (33)	1.0 (0.9-1.3)	176/547 (32)	1.0 (0.8-1.2)	24/62 (39)	1.2 (0.8-1.7)
Leakage of solid stools while asleep at least occasionally	15/611 (2)	0/344 (0)	-	12/548 (2)	-	3/63 (5)	-
Bowel symptoms							
Defaecation urgency at least once a week	175/602 (29)	19/341 (6)	**5.7 (3.5-9.1)**	157/542 (29)	**5.7 (3.5-9.1)**	18/60 (30)	**6.0 (3.3-10.8)**
Protracted abdominal pain lasting more than 1 year, yes	69/593 (12)	15/339 (4)	**3.2 (1.9-5.6)**	59/532 (11)	**3.1 (1.7-5.5)**	10/61 (16)	**3.8 (1.8-7.9)**
Loose stools at least once a week	234/602 (39)	48/344 (14)	**3.0 (2.2-3.9)**	205/540 (38)	**2.9 (2.1-3.9)**	29/62 (47)	**3.5 (2.4-5.1)**
Abdominal pain and vomiting at least occasionally	60/605 (10)	13/344 (4)	**2.6 (1.4-4.7)**	49/545 (9)	**2.2 (1.2-4.1)**	11/60 (18)	**4.8 (2.2-10.1)**
Mucus in stools at least occasionally	156/607 (26)	45/343 (13)	**2.1 (1.5-3.0)**	143/545 (26)	**2.2 (1.6-3.0)**	13/62 (21)	1.6 (0.9-2.8)
Abdominal bloating at least once a week	147/605 (24)	60/342 (18)	**1.8 (1.4-2.3)**	129/544 (24)	**1.8 (1.3-2.3)**	18/61 (30)	**1.8 (1.1-2.7)**
Rectal bleeding at least occasionally	103/604 (17)	42/341(12)	**1.6 (1.1-2.2)**	92/545 (17)	**1.6 (1.2-2.4)**	11/59 (19)	1.5 (0.8-2.8)
Abdominal pain and stools at least occasionally	198/602 (33)	79/343 (23)	**1.6 (1.3-2.0)**	171/541 (32)	**1.5 (1.2-1.9)**	27/61 (44)	**2.0 (1.4-2.8)**

Appendix Table A3. Late Symptoms during the Past Six Months among Gynaecological Cancer Survivors Treated with Pelvic Radiation Therapy with or without Surgery and Control Women, all symptoms

	**Survivors** *N=616 (%)*	**Controls** *N=344 (%)*	**Survivors vs. Controls Age adjusted RR (95% CI)**	**RT with Surgery** *N=549 (%)*	**RT with Surgery vs. Controls Age adjusted RR (95% CI)**	**RT without Surgery** *N= 67 (%)*	**RT without Surgery vs. Controls Age adjusted RR (95% CI)**
Bowel symptoms continued							
Abdominal pain at least occasionally	307/604 (51)	137/340 (40)	**1.4 (1.2-1.6)**	272/544 (50)	**1.4 (1.2-1.6)**	35/60 (58)	**1.5 (1.2-1.9)**
Abdominal pain and bloating at least occasionally	228/603 (38)	106/340(31)	**1.4 (1.2-1.7)**	199/542 (37)	**1.4 (1.2-1.7)**	29/61 (48)	**1.6 (1.2-2.1)**
Incomplete bowel emptying at least occasionally	296/609 (49)	152/340 (45)	1.1 (1.0-1.3)	273/549 (50)	1.2 (1.0-1.4)	23/60 (38)	0.9 (0.6-1.2)
Ability to exert strain at stool, at least moderately good	187/604 (31)	108/338 (32)	1.0 (0.8-1.2)	176/543 (32)	1.1 (0.9-1.3)	11/61 (18)	0.6 (0.3-1.0)
Hard stools at least once a week	40/608 (7)	32/344 (9)	0.7 (0.5-1.2)	38/546 (7)	0.8 (0.5-1.3)	2/62 (3)	0.3 (0.1-1.4)
Urinary tract symptoms							
Difficulty feeling the need to empty bladder at least occasionally	56/604 (9)	11/343 (3)	**2.8 (1.5-5.4)**	46/544 (8)	**2.5 (1.3-5.0)**	10/60 (17)	**5.1 (2.3-11.3)**
Difficulty emptying bladder at least occasionally	49/602 (8)	11/343 (3)	**2.7 (1.4-5.2)**	43/542 (8)	**2.7 (1.4-5.5)**	6/60 (10)	**3.2 (1.3-8.4)**
Difficulty feeling full bladder at least occasionally	90/602 (15)	18/342 (5)	**2.7 (1.6-4.5)**	76/543 (14)	**2.4 (1.4-4.1)**	14/59 (24)	**4.5 (2.4-8.4)**
Haematuria at least occasionally	20/606 (3)	5/344 (1)	2.5 (0.9-6.9)	16/546 (3)	2.4 (0.8-6.8)	4/60 (7)	**5.0 (1.4-17.8)**
Straining to initiate emptying of bladder at least occasionally	77/605 (13)	21/343 (6)	**2.2 (1.4-3.7)**	68/545 (12)	**2.4 (1.5-3.9)**	9/60 (15)	**2.5 (1.2-5.3)**
Painful emptying of bladder at least occasionally	60/605 (10)	20/341 (6)	**2.0 (1.2-3.3)**	52/545 (10)	**1.9 (1.1-3.3)**	8/60 (13)	**2.3 (1.1-5.0)**
Urinary incontinence without urinary urgency at least occasionally	88/603 (15)	24/342 (7)	**1.8 (1.2-2.9)**	81/543 (15)	**1.9 (1.2-3.0)**	7/60 (12)	1.6 (0.7-3.6)
Night-time emptying of bladder at least twice per night or more, yes	222/607 (37)	58/341 (17)	**1.8 (1.4-2.4)**	199/548 (36)	**1.8 (1.4-2.4)**	23/59 (39)	**2.2 (1.5-3.2)**
Need of antibiotics due to urinary tract infection twice or more, yes	93/605 (15)	31/344 (9)	**1.6 (1.1-2.3)**	86/545 (16)	**1.7 (1.1-2.5)**	7/60 (12)	1.3 (0.6-2.9)
Urinary incontinence due to urinary urgency at least occasionally	209/607 (34)	66/342 (19)	**1.6 (1.2-2.0)**	186/545 (34)	**1.5 (1.2-2.0)**	23/62 (37)	**1.8 (1.2-2.7)**
Slow emptying of bladder at least occasionally	122/605 (20)	47/344 (14)	**1.6 (1.2-2.2)**	106/546 (19)	**1.5 (1.1-2.2)**	16/59 (27)	**2.0 (1.2-3.3)**
Self-perception of urine odor at least occasionally	149/605 (25)	62/341(18)	**1.4 (1.1-1.9)**	126/544 (23)	**1.3 (1.0-1.8)**	23/61 (38)	**2.1 (1.4-3.0)**
Urinary urgency at least occasionally	346/605 (57)	139/343 (41)	**1.3 (1.1-1.5)**	308/544 (57)	**1.3 (1.1-1.5)**	38/61 (62)	**1.5 (1.2-1.8)**
Feeling of incomplete bladder emptying at least occasionally	232/604 (38)	101/343 (29)	**1.3 (1.1-1.6)**	203/544 (37)	**1.3 (1.0-1.6)**	29/60 (48)	**1.6 (1.2-2.2)**

Appendix Table A3. Late Symptoms during the Past Six Months among Gynaecological Cancer Survivors Treated with Pelvic Radiation Therapy with or without Surgery and Control Women, all symptoms

	**Survivors** *N=616 (%)*	**Controls** *N=344 (%)*	**Survivors vs. Controls Age adjusted RR (95% CI)**	**RT with Surgery** *N=549 (%)*	**RT with Surgery vs. Controls Age adjusted RR (95% CI)**	**RT without Surgery** *N= 67 (%)*	**RT without Surgery vs. Controls Age adjusted RR (95% CI)**
Symptoms related to sexuality							
Protracted genital pain lasting for more than 1 year yes	28/593 (5)	4/339 (1)	**5.0 (1.7-14.5)**	21/532 (4)	**4.3 (1.4-13.0)**	7/61 (11)	**9.6 (2.9-31.8)**
Genital bleeding during or after intercourse at least once, yes	54/585 (9)	13/329 (4)	**3.7 (2.1-6.7)**	45/523 (9)	**3.6 (2.0-6.7)**	9/62 (15)	**3.9 (1.8-8.4)**
Deep dyspareunia when having intercourse, at least a little	101/583 (17)	23/330 (7)	**3.7 (2.4-5.7)**	90/521 (17)	**3.7 (2.4-5.8)**	11/62 (18)	**3.0 (1.6-5.7)**
Vaginal lubrication when sexually aroused, no	39/577 (7)	7/333 (2)	**2.9 (1.3-6.4)**	37/517 (7)	**2.9 (1.3-6.5)**	2/60 (3)	1.6 (0.3-7.5)
Decreased ability for intercourse leading to lower intercourse frequency, at least a little	176/575 (31)	37/327 (11)	**2.9 (2.1-4.1)**	160/515 (31)	**2.9 (2.0-4.0)**	11/60 (27)	**2.5 (1.5-4.1)**
Vaginal elasticity, no	172/502 (34)	41/302 (14)	**1.8 (1.3-2.4)**	148/451 (33)	**1.6 (1.2-2.2)**	24/51 (47)	**2.5 (1.7-3.7)**
Genital swelling when sexually aroused, no	82/571 (14)	35/327 (11)	**1.5 (1.0-2.2)**	72/512 (14)	1.5 (1.0-2.2)	10/59 (17)	1.6 (0.9-3.1)
Superficial dyspareunia when having intercourse, at least a little	140/584 (24)	67/330 (20)	**1.5 (1.2-2.0)**	125/523 (24)	**1.5 (1.1-2.0)**	15/61 (25)	1.4 (0.8-2.2)
Sexual arousal in a sexual situation, no	55/578 (10)	20/331 (6)	1.3 (0.8-2.1)	50/516 (10)	1.3 (0.8-2.2)	5/62 (8)	1.3 (0.5-3.2)
Sensitivity to touch inside vagina, no	221/540 (41)	65/310 (21)	**1.3 (1.0-1.6)**	192/485 (40)	1.2 (1.0-1.5)	29/55 (53)	**1.7 (1.3-2.2)**
Sensitivity to touch of labia and clitoris, no	186/558 (33)	61/320 (19)	1.2 (0.9-1.5)	163/501 (33)	1.1 (0.9-1.4)	23/57 (40)	**1.5 (1.1-2.1)**
Chafing of labia, yes	117/573 (20)	71/329 (22)	1.1 (0.8-1.4)	103/514 (20)	1.1 (0.8-1.4)	14/59 (24)	1.2 (0.7-1.9)
Orgasm, no	294/565 (52)	98/323 (30)	1.1 (1.0-1.3)	265/507 (52)	1.1 (0.9-1.3)	29/58 (50)	1.1 (1.0-1.4)
Pelvic bone symptoms							
Pubic pain when walking indoors at least occasionally	46/603 (8)	6/343 (2)	**4.9 (2.1-11.6)**	35/542 (6)	**4.1 (1.7-10.2)**	11/61 (18)	**10.3 (4.0-26.7)**
Pubic pain when walking outdoors 500 m at least occasionally	42/596 (7)	7/343 (2)	**3.7 (1.7-8.4)**	34/536 (6)	**3.3 (1.4-7.7)**	8/60 (13)	**6.6 (2.5-17.5)**
Pubic pain, yes	67/603 (11)	12/339 (4)	**3.4 (1.9-6.4)**	53/544 (10)	**3.0 (1.6-5.7)**	14/59 (24)	**7.0 (3.5-14.2)**
Protracted hip pain lasting for more than 1 year, yes	144/593 (24)	65/339 (19)	1.2 (0.9-1.5)	127/521 (24)	1.1 (0.8-1.5)	17/61 (28)	1.5 (1.0-2.3)
Hip pain when walking indoors at least occasionally	191/598 (32)	97/341 (28)	1.1 (0.9-1.3)	167/538 (31)	1.0 (0.8-1.3)	24/60 (40)	1.4 (1.0-2.0)
Protracted back pain lasting for more than 1 year, yes	180/593 (30)	96/339 (28)	1.1 (0.9-1.3)	161/532 (30)	1.1 (0.8-1.3)	19/61 (31)	1.1 (0.7-1.6)
Hip pain, yes	212/599 (35)	113/343 (33)	1.0 (0.8-1.2)	182/538 (34)	0.9 (0.8-1.2)	30/61 (49)	**1.5 (1.1-2.0)**
Hip pain when walking outdoors 500 m at least occasionally	193/598 (32)	102/340 (30)	1.0 (0.8-1.3)	165/536 (31)	1.0 (0.8-1.2)	28/62 (45)	**1.5 (1.1-2.0)**
Protracted joint pain lasting for more than 1 year, yes	126/593 (21)	64/339 (19)	1.0 (0.8-1.4)	113/532 (21)	1.0 (0.8-1.4)	13/61 (21)	1.1 (0.7-1.9)

Appendix Table A3. Late Symptoms during the Past Six Months among Gynaecological Cancer Survivors Treated with Pelvic Radiation Therapy with or without Surgery and Control Women, all symptoms

	**Survivors** *N=616 (%)*	**Controls** *N=344 (%)*	**Survivors vs. Controls Age adjusted RR (95% CI)**	**RT with Surgery** *N=549 (%)*	**RT with Surgery vs. Controls Age adjusted RR (95% CI)**	**RT without Surgery** *N= 67 (%)*	**RT without Surgery vs. Controls Age adjusted RR (95% CI)**
Pelvic bone symptoms continued							
Sacral pain when walking outdoors 500 m at least occasionally	190/584 (33)	119/343 (35)	0.9 (0.7-1.1)	166/524 (32)	0.9 (0.7-1.1)	24/60 (40)	1.1 (0.8-1.6)
Sacral pain, yes	232/600 (39)	179/344 (52)	**0.8 (0.7-0.9)**	196/539 (36)	**0.7 (0.6-0.8)**	36/61 (59)	1.1 (0.9-1.4)
Sacral pain when walking indoors at least occasionally	194/593 (33)	134/342 (39)	0.8 (0.7-1.0)	166/531 (31)	0.8 (0.6-1.0)	28/62 (45)	1.2 (0.9-1.6)
Lower abdomen and leg symptoms							
Erysipelas on abdomen or legs, yes	17/597 (3)	3/336 (1)	**3.6 (1.0-12.8)**	14/537 (3)	**4.0 (1.1-14.5)**	3/60 (5)	**5.2 (1.1-25.1)**
Lower abdominal heaviness at least occasionally	119/600 (20)	38/344 (11)	**2.1 (1.5-3.0)**	101/540 (19)	**2.1 (1.4-3.0)**	18/60 (30)	**2.7 (1.7-4.5)**
Pain in lower abdomen in connection with edema at least occasionally	92/607 (15)	33/343 (10)	**1.9 (1.3-2.8)**	75/546 (14)	**1.7 (1.1-2.6)**	17/61 (28)	**3.0 (1.8-5.0)**
Leg pain in connection with edema at least occasionally	164/606 (27)	54/342 (16)	**1.7 (1.3-2.3)**	145/545 (27)	**1.7 (1.2 (2.2)**	19/61 (31)	**2.0 (1.3-3.2)**
Swollen lower abdomen at least occasionally	121/599 (20)	51/342 (15)	**1.7 (1.3-2.4)**	106/538 (20)	**1.8 (1.3-2.5)**	15/61 (25)	**1.7 (1.0-2.8)**
Protracted leg pain lasting more than 1 year, yes	134/593 (21)	48/339 (14)	**1.5 (1.1-2.1)**	115/532 (22)	**1.4 (1.0-2.0)**	19/61 (31)	**2.2 (1.4-3.4)**
Swollen legs at least occasionally	218/606 (36)	94/344 (27)	**1.4 (1.1-1.7)**	197/546 (36)	**1.4 (1.1-1.9)**	21/60 (35)	1.4 (0.9-2.0)
Heavy legs at least occasionally	210/606 (35)	97/344 (28)	**1.1 (1.1-1.6)**	186/546 (34)	**1.3 (1.0-1.6)**	24/60 (40)	**1.4 (1.0-2.1)**

Abbreviations: RR = relative risk; CI = confidence interval; RT = radiation therapy

The symptoms are sorted into anatomical region of supposed origin and in size order for relative risk, the number in the denominator may vary due to missing information
