# Supplementary material for: Late symptoms in long-term gynaecological cancer survivors after radiation therapy: a population-based cohort study
Source: Br J Cancer. 2011 Aug 16;105(6):737–45. doi: 10.1038/bjc.2011.315 (PMC3171018; doi:10.1038/bjc.2011.315)
Supplement: Supplementary Appendix Table A4 [file bjc2011315x8.pdf]

Appendix Table A4. Late Symptoms during the Past Six Months among Gynaecological Cancer Survivors in Relation to Diagnosis

	**Endometrial Cancer**	**Cervical Cancer**		**Ovarian and Fallopian Tube Cancer**	**Sarcoma Uteri**	**Vaginal Cancer**		**Vulvar Cancer**
		No surgery	***Surgery***			***No surgery***	***Surgery***	
	N=366 (%)	*N=56 (%)*	*N=86 (%)*	*N=58 (%)*	*N=30 (%)*	*N=7 (%)*	*N=7 (%)*	*N=6 (%)*
Anal-sphincter symptoms								
Emptying of all stools into clothing without forewarning at least occasionally	32/361 (9)	13/54 (24)	8/86 (9)	5/58 (9)	9/29 (31)	2/6 (33)	0/7 (0)	1/5 (20)
Leakage of loose stools while awake at least occasionally	104/360 (29)	23/56 (41)	25/86 (29)	23/58 (40)	16/29 (55)	5/7 (71)	1/7 (14)	2/5 (40)
Leakage of loose stools while asleep at least occasionally	34/363 (9)	12/56 (21)	10/86 (12)	6/58 (10)	6/29 (21)	2/7 (29)	1/7 (14)	1/5 (20)
Anal leakage of mucus while asleep at least occasionally	11/361 (3)	5/55 (9)	7/86 (8)	3/58 (5)	3/28 (11)	2/7 (29)	1/7 (14)	0/5 (0)
Leakage of solid stools while awake at least occasionally	23/359 (6)	7/56 (12)	6/86 (7)	4/58 (7)	1/29 (3)	2/7 (29)	1/7 (14)	2/5 (40)
Feacal leakage without forewarning despite previous defeacation at least occasionally	107/360 (30)	18/54 (33)	24/86 (28)	20/58 (34)	14/29 (48)	2/6 (33)	2/7 (29)	1/5 (20)
Defeacation urgency with feacal leakage at least occasionally	165/358 (46)	32/55 (58)	39/84 (46)	31/58 (53)	21/29 (72)	4/7 (57)	4/7 (57)	2/5 (40)
Foul smelling flatulence at least once a week	57/356 (16)	12/55 (22)	22/86 (26)	14/58 (24)	7/29 (24)	2/6 (33)	1/7 (14)	1/5 (20)
Anal leakage of mucus while awake at least occasionally	40/358 (11)	8/55 (15)	16/86 (19)	10/58 (17)	8/28 (29)	3/6 (50)	2/7 (29)	0/5 (0)
Self-perception of feacal odor at least occasionally	59/359 (16)	11/56 (20)	14/86 (16)	10/58 (17)	9/29 (31)	2/6 (33)	2/7 (29)	1/5 (20)
Anal leakage of blood while asleep at least occasionally	5/361 (1)	1/55 (2)	1/86 (1)	0/58 (0)	2/29 (7)	0/7 (0)	0/7 (0)	0/5 (0)
Involuntary flatulence at least once a week	63/360 (18)	15/55 (27)	21/86 (24)	16/58 (28)	8/29 (28)	1/6 (17)	1/7 (14)	2/5 (40)
Unwanted defeacation while emptying bladder at least occasionally	131/357 (37)	23/55 (42)	39/85 (46)	24/57 (42)	14/29 (48)	2/7 (29)	2/7 (29)	3/6 (50)
Anal leakage of blood while awake at least occasionally	23/360 (6)	4/56 (7)	6/86 (7)	4/58 (7)	3/29 (10)	1/7 (14)	0/7 (0)	1/5 (20)
Involuntary loud flatulence at least occasionally	199/361 (55)	40/55 (73)	51/86 (59)	37/58 (64)	21/29 (72)	5/6 (83)	3/7 (43)	3/5 (60)
Anal pain at least occasionally	70/363 (19)	9/54 (17)	21/86 (24)	16/58 (28)	6/29 (21)	4/6 (67)	2/7 (29)	1/5 (20)
Pruritus ani at least occasionally	105/362 (29)	20/55 (36)	31/86 (36)	26/58 (45)	10/29 (35)	4/7 (57)	3/7 (43)	1/5 (20)
Leakage of solid stools while asleep at least occasionally	6/363 (2)	3/56 (5)	3/86 (4)	2/58 (3)	0/29 (0)	0/7 (0)	1/7 (14)	0/5 (0)
Bowel symptoms								
Defeacation urgency at least once a week	101/360 (28)	15/54 (28)	26/84 (31)	16/57 (28)	11/29 (38)	3/6 (50)	2/7 (29)	1/5 (20)
Protracted abdominal pain lasting more than 1 year, yes	27/350 (8)	8/54 (15)	17/82 (21)	6/58 (10)	6/29 (21)	2/7 (29)	1/7 (14)	2/6 (33)
Loose stools at least once a week	125/356 (35)	25/55 (45)	33/84 (39)	25/57 (44)	16/30 (53)	4/7 (57)	5/7 (71)	1/6 (17)
Abdominal pain and vomiting at least occasionally	31/363 (9)	10/53 (19)	10/84 (12)	4/58 (7)	2/28 (7)	1/7 (14)	1/7 (14)	1/5 (20)
Mucus in stools at least occasionally	79/361 (22)	10/55 (18)	29/86 (34)	18/58 (31)	11/28 (39)	3/7 (43)	4/7 (57)	2/5 (40)
Abdominal bloating at least once a week	66/361 (18)	15/54 (28)	34/84 (40)	16/58 (28)	8/29 (28)	3/7 (43)	3/7 (43)	2/5 (40)

Appendix Table A4. Late Symptoms during the Past Six Months among Gynaecological Cancer Survivors in Relation to Diagnosis

	**Endometrial Cancer**	**Cervical Cancer**		**Ovarian and Fallopian Tube Cancer**	**Sarcoma Uteri**	**Vaginal Cancer**		**Vulvar Cancer**
		No surgery	***Surgery***			***No surgery***	***Surgery***	
	N=366 (%)	*N=56 (%)*	*N=86 (%)*	*N=58 (%)*	*N=30 (%)*	*N=7 (%)*	*N=7 (%)*	*N=6 (%)*
Bowel symptoms continued								
Rectal bleeding at least occasionally	53/361 (15)	10/52 (19)	19/85 (22)	8/58 (14)	6/29 (21)	1/7 (14)	3/7 (43)	3/5 (60)
Abdominal pain and stools at least occasionally	102/359 (28)	23/54 (43)	35/84 (42)	19/58 (33)	14/28 (50)	4/7 (57)	1/7 (14)	0/5 (0)
Abdominal pain at least occasionally	168/362 (46)	31/53 (59)	54/84 (64)	29/58 (50)	17/28 (61)	4/7 (57)	2/7 (29)	2/5 (40)
Abdominal pain and bloating at least occasionally	114/362 (32)	25/54 (46)	45/84 (54)	24/58 (41)	13/27 (48)	4/7 (57)	1/7 (14)	2/5 (40)
Incomplete bowel emptying at least occasionally	162/364 (45)	18/53 (34)	52/86 (60)	32/57 (56)	19/30 (63)	5/7 (71)	6/7 (86)	2/5 (40)
Ability to exert strain at stool at least moderately good	100/358 (28)	10/54 (19)	42/86 (49)	19/57 (33)	10/30 (33)	1/7 (14)	3/7 (43)	2/5 (40)
Hard stools at least once a week	17/359 (5)	2/55 (4)	11/86 (13)	8/58 (14)	2/30 (7)	0/7 (0)	0/7 (0)	0/6 (0)
Urinary tract symptoms								
Difficulty feeling the need to empty bladder at least occasionally	24/362 (7)	9/53 (17)	14/83 (17)	4/58 (7)	4/28 (14)	1/7 (14)	0/7 (0)	0/6 (0)
Difficulty emptying bladder at least occasionally	23/361 (6)	6/54 (11)	11/83 (13)	7/56 (12)	1/29 (3)	0/6 (0)	0/7 (0)	1/6 (17)
Difficulty feeling full bladder at least occasionally	47/361 (13)	14/53 (26)	16/83 (19)	5/58 (9)	6/29 (21)	0/6 (0)	1/7 (14)	1/5 (20)
Haematuria at least occasionally	27/339 (8)	2/54 (4)	4/83 (5)	3/54 (6)	1/29 (3)	0/6 (0)	0/7 (0)	2/5 (40)
Straining to initiate emptying of bladder at least occasionally	34/362 (9)	8/54 (15)	22/83 (27)	8/58 (14)	2/29 (7)	1/6 (17)	2/7 (29)	0/6 (0)
Painful emptying of bladder at least occasionally	26/362 (7)	6/54 (11)	11/84 (13)	8/58 (14)	3/28 (11)	2/6 (33)	2/7 (29)	2/6 (33)
Urinary incontinence without urinary urgency at least occasionally	56/358 (16)	7/54 (13)	11/85 (13)	4/58 (7)	7/29 (24)	0/6 (0)	1/7 (14)	2/6 (33)
Night-time emptying of bladder at least twice per night or more, yes	137/363 (38)	22/54 (41)	27/85 (32)	17/58 (29)	15/29 (52)	1/5 (20)	2/7 (29)	1/6 (17)
Need of antibiotics due to urinary tract infection twice or more, yes	53/360 (15)	6/54 (11)	17/85 (20)	7/58 (12)	7/29 (24)	1/6 (17)	1/7 (14)	1/6 (17)
Urinary incontinence due to urinary urgency at least occasionally	119/360 (33)	21/55 (38)	28/85 (33)	18/58 (31)	11/29 (38)	2/7 (29)	5/7 (71)	5/6 (83)
Slow emptying of bladder at least occasionally	56/361 (16)	14/54 (26)	23/85(27)	15/58 (26)	9/29 (31)	2/5 (40)	2/7 (29)	1/6 (17)
Self-perception of urine odor at least occasionally	77/359 (21)	23/55 (42)	24/85 (28)	12/58 (21)	9/29 (31)	0/6 (0)	2/7 (29)	2/6 (33)
Urinary urgency at least occasionally	203/359 (57)	33/54 (61)	45/85 (53)	30/58 (52)	19/29 (66)	5/7 (71)	6/7 (86)	5/6 (83)
Feeling of incomplete bladder emptying at least occasionally	123/360 (34)	27/54 (50)	40/84 (48)	22/58 (38)	11/29 (38)	2/6 (33)	4/7 (57)	3/6 (50)

Appendix Table A4. Late Symptoms during the Past Six Months among Gynaecological Cancer Survivors in Relation to Diagnosis

	Endometrial Cancer	Cervical Cancer		Ovarian and Fallopian Tube Cancer	Sarcoma Uteri	Vaginal Cancer		Vulvar Cancer
		No surgery	*Surgery*			*No surgery*	*Surgery*	
	N=366 (%)	*N=56 (%)*	*N=86 (%)*	*N=58 (%)*	*N=30 (%)*	*N=7 (%)*	*N=7 (%)*	*N=6 (%)*
Symptoms related to sexuality								
Protracted genital pain lasting for more than 1 year, yes	12/350 (3)	4/54 (7)	5/82 (6)	1/58 (2)	1/29 (3)	3/7 (43)	1/7 (14)	1/6 (17)
Genital bleeding during or after intercourse at least once,	24/345 (7)	8/55 (15)	18/83 (22)	0/54 (0)	3/29 (10)	1/7 (14)	0/7 (0)	0/5 (0)
Deep dyspareunia when having intercourse, yes	48/344 (14)	9/55 (16)	27/82 (33)	8/54 (15)	4/29 (14)	2/7 (29)	2/7 (29)	1/5 (20)
Vaginal lubrication when sexually aroused, no	27/339 (8)	2/54 (4)	4/83 (5)	3/54 (6)	1/29 (3)	0/6 (0)	0/7 (0)	2/5 (40)
Decreased ability for intercourse leading to lower intercourse frequency, yes	104/339 (31)	15/54 (28)	31/84 (37)	14/54 (26)	5/27 (19)	1/6 (17)	5/6 (83)	1/5 (20)
Vaginal elasticity, no	112/290 (39)	21/45 (47)	16/75 (21)	11/50 (22)	6/25 (24)	3/6 (50)	1/7 (14)	2/4 (50)
Genital swelling when sexually aroused, no	47/336 (14)	8/53 (15)	14/83 (17)	6/53 (11)	3/28 (11)	2/6 (33)	0/7 (0)	2/5 (40)
Superficial dyspareunia when having intercourse, at least a little	70/345 (20)	13/54 (24)	31/82 (38)	14/55 (25)	5/29 (17)	2/7 (29)	3/7 (43)	2/5 (40)
Sexual arousal in a sexual situation, no	33/338 (10)	4/55 (7)	8/83 (10)	4/54 (7)	3/29 (10)	1/7 (14)	0/7 (0)	2/5 (40)
Sensitivity to touch inside vagina, no	148/318 (47)	25/49 (51)	21/79 (27)	14/51 (27)	7/25 (28)	4/6 (67)	1/7 (14)	1/5 (20)
Sensitivity to touch of labia and clitoris, no	126/329 (38)	20/51 (39)	17/81 (21)	12/53 (23)	5/26 (19)	3/6 (50)	1/7 (14)	2/5 (40)
Chafing of labia, yes	58/339 (17)	10/53 (19)	19/81 (23)	16/54 (30)	5/28 (18)	4/6 (67)	3/7 (43)	2/5 (40)
Orgasm, no	198/334 (59)	26/52 (50)	28/80 (35)	24/53 (45)	10/28 (36)	3/6 (50)	2/7 (29)	3/5 (60)
Pelvic bone symptoms								
Pubic pain when walking indoors at least occasionally	18/360 (5)	7/54 (13)	7/85 (8)	7/56 (12)	1/28 (4)	4/7 (57)	0/7 (0)	2/6 (33)
Pubic pain when walking outdoors 500 m at least occasionally	18/356 (5)	7/54 (13)	6/83 (7)	7/56 (12)	1/28 (4)	1/6 (17)	0/7 (0)	2/6 (33)
Pubic pain, yes	29/361 (8)	10/53 (19)	10/85 (12)	8/56 (14)	2/29 (7)	4/6 (67)	2/7 (29)	2/6 (33)
Protracted hip pain lasting for more than 1 year, yes	87/350 (25)	14/54 (26)	14/82 (17)	12/58 (21)	12/29 (41)	3/7 (43)	1/7 (14)	1/6 (17)
Hip pain when walking indoors at least occasionally	103/357 (29)	19/53 (36)	24/85 (28)	23/55 (42)	11/28 (39)	5/7 (71)	3/7 (43)	3/6 (50)
Protracted back pain lasting for more than 1 year, yes	97/350 (28)	16/54 (30)	28/82 (34)	19/58 (33)	11/29 (38)	3/7 (43)	4/7 (57)	2/6 (33)
Hip pain, yes	111/356 (31)	25/55 (45)	31/84 (37)	23/57 (40)	11/28 (39)	5/6 (83)	3/7 (43)	3/6 (50)
Hip pain when walking outdoors 500 m at least occasionally	101/355 (28)	24/55 (44)	26/85 (31)	22/55 (40)	11/28 (39)	4/7 (57)	2/7 (29)	3/6 (50)
Protracted joint pain lasting for more than 1 year, yes	76/350 (22)	10/54 (19)	15/82 (18)	14/58 (24)	5/29 (17)	3/7 (43)	1/7 (14)	2/6 (33)
Sacral pain when walking outdoors 500 m at least occasionally	106/351 (30)	21/53 (40)	22/82 (27)	20/52 (38)	13/28 (46)	3/7 (43)	4/7 (57)	1/4 (25)

Pelvic bone symptoms continued

Appendix Table A4. Late Symptoms during the Past Six Months among Gynaecological Cancer Survivors in Relation to Diagnosis

	Endometrial Cancer	Cervical Cancer		Ovarian and Fallopian Tube Cancer	Sarcoma Uteri	Vaginal Cancer		Vulvar Cancer
		No surgery	*Surgery*			*No surgery*	*Surgery*	
	N=366 (%)	*N=56 (%)*	*N=86 (%)*	*N=58 (%)*	*N=30 (%)*	*N=7 (%)*	*N=7 (%)*	*N=6 (%)*
Sacral pain, yes	118/357 (33)	32/54 (59)	32/84 (38)	25/58 (43)	14/28 (50)	4/7 (57)	5/7 (71)	2/5 (40)
Sacral pain when walking indoors at least occasionally	110/353 (31)	24/55 (44)	24/84 (29)	17/54 (31)	8/28 (29)	4/7 (57)	5/7 (71)	2/5 (40)
Lower abdomen and leg symptoms								
Erysipelas on abdomen or legs, yes	9/357 (3)	3/54 (6)	5/83 (6)	0/57 (0)	0/28 (0)	0/6 (0)	0/7 (0)	0/5 (0)
Lower abdominal heaviness at least occasionally	54/356 (15)	16/54 (30)	31/84 (37)	8/58 (14)	4/29 (14)	2/6 (33)	0/7 (0)	4/6 (67)
Pain in lower abdomen in connection with edema at least occasionally	43/362 (12)	15/54 (28)	19/84 (23)	6/58 (10)	4/29 (14)	2/7 (29)	0/7 (0)	3/6 (50)
Leg pain in connection with edema at least occasionally	90/361 (25)	17/54 (32)	27/84 (32)	15/58 (26)	7/29 (24)	2/7 (29)	1/7 (14)	5/6 (83)
Swollen legs at least occasionally	114/362 (31)	18/54 (33)	48/85 (57)	20/58 (34)	10/28 (36)	3/6 (50)	1/7 (14)	4/6 (67)
Protracted leg pain lasting more than 1 year, yes	76/350 (22)	15/54 (28)	21/82 (26)	9/58 (16)	5/29 (17)	4/7 (57)	1/7 (14)	3/6 (50)
Swollen lower abdomen at least occasionally	54/358 (15)	15/55 (27)	36/84 (43)	10/57 (18)	2/27 (7)	0/6 (0)	1/7 (14)	3/5 (60)
Heavy legs at least occasionally	107/362 (30)	22/54 (41)	48/85 (56)	17/58 (29)	8/28 (29)	2/6 (33)	1/7 (14)	5/6 (83)

The symptoms are sorted in anatomical region of supposed origin, the number in the denominator may vary due to missing information
