# Supplementary material for: Late symptoms in long-term gynaecological cancer survivors after radiation therapy: a population-based cohort study
Source: Br J Cancer. 2011 Aug 16;105(6):737–45. doi: 10.1038/bjc.2011.315 (PMC3171018; doi:10.1038/bjc.2011.315)

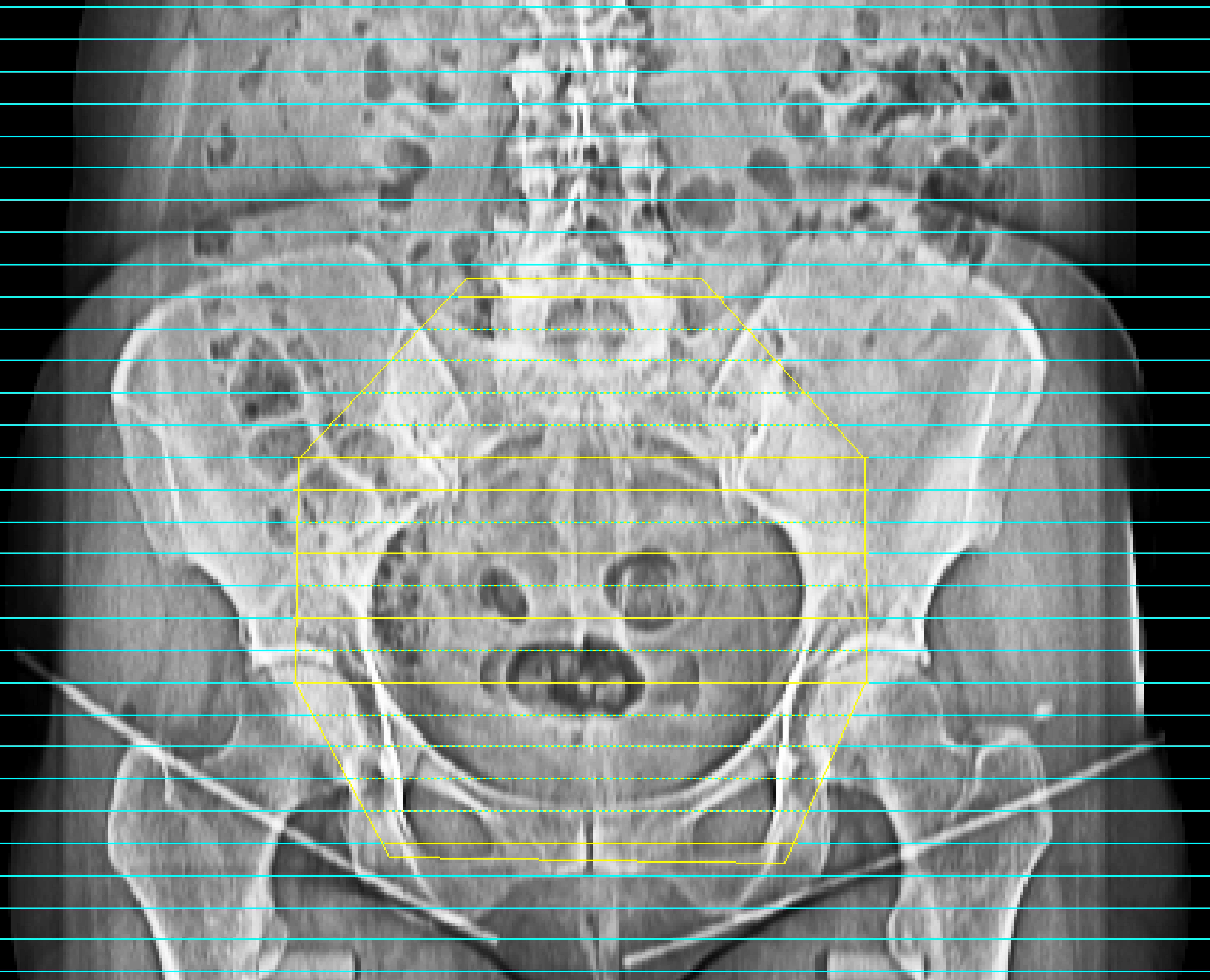

Supplement: Supplementary Appendix Figure A1 [file bjc2011315x1.pdf]
